# Plasma neurofilament light and its association with all-cause mortality risk among urban middle-aged men and women

**DOI:** 10.1186/s12916-022-02425-x

**Published:** 2022-06-13

**Authors:** May A. Beydoun, Nicole Noren Hooten, Jordan Weiss, Hind A. Beydoun, Sharmin Hossain, Michele K. Evans, Alan B. Zonderman

**Affiliations:** 1 Laboratory of Epidemiology and Population Sciences, NIA/NIH/IRP, 251 Bayview Blvd., Suite 100, Room #: 04B118, Baltimore, MD 21224 USA; 2 Department of Demography, University of California, Berkeley, Berkeley, CA USA; 3 Department of Research Programs, Fort Belvoir Community Hospital, Fort Belvoir, VA USA

**Keywords:** Biomarker, Death, Humans, Race, NfL, Plasma, Prognosis

## Abstract

**Background:**

Neurofilament light chain (NfL) is released into the blood during neuronal damage. NfL is linked to mortality in neurological disorders, remaining unexplored in population studies. We investigated whether initial (v_1_) and annualized change (*δ*) in plasma NfL can predict all-cause mortality in middle-aged dementia-free urban adults.

**Methods:**

Longitudinal data were from 694 participants in the Healthy Aging in Neighborhoods of Diversity Across the Life Span study (HANDLS, mean age_v1_: 47.8 years, 42% male, 55.8% African American). Plasma NfL was measured prospectively at three visits. Analyses included Cox proportional hazards models for all-cause mortality risk and 4-way decomposition testing for interaction and mediation.

**Results:**

Unlike men, women exhibited a direct association between δNfL (above vs. below median) and all-cause mortality risk in both the minimally (HR = 3.91, 95% CI 1.10–13.9, *p* = 0.036) and fully adjusted models (HR = 4.92, 95% CI 1.26–19.2, *p* = 0.022), and for δNfL (per unit increase) in the full model (HR = 1.65, 95% CI 1.04–2.61, *p* = 0.034). In both models, and among women, 1 standard deviation of NfL_v1_ was associated with an increased all-cause mortality risk (reduced model: HR = 2.01, 95% CI 1.24–3.25, *p* = 0.005; full model: HR = 1.75, 95% CI 1.02–2.98, *p* = 0.041). Only few interactions were detected for cardio-metabolic risk factors. Notably, NfL_v1_ was shown to be a better prognostic indicator at normal hsCRP values among women, while HbA1c and δNfL interacted synergistically to determine mortality risk, overall.

**Conclusions:**

These findings indicate that plasma NfL levels at baseline and over time can predict all-cause mortality in women and interacts with hsCRP and HbA1c to predict that risk.

**Supplementary Information:**

The online version contains supplementary material available at 10.1186/s12916-022-02425-x.

## Background

Neurofilament light chain (NfL) is a cytoskeletal protein component exclusively expressed in neurons that is released into the extracellular fluids, including blood, during neuroaxonal damage. Emerging data indicates the clinical utility of blood-based measurements of NfL as a novel biomarker for neurodegenerative diseases. This methodological development for assaying plasma NfL has stimulated opportunities for large-scale applications in clinical practice and in randomized clinical trials as a method for identifying patients at risk for dementias, including Alzheimer’s disease (AD) [1]. Thus far, NfL reflects sub-cortical large-caliber axonal degeneration [2, 3]. Plasma NfL levels correlate strongly with cerebrospinal fluid (CSF) NfL levels [1, 4], adding to its clinical utility in differential diagnoses for dementias [5–8] and other neurodegenerative diseases [9–12]. Therefore, plasma NfL measurements are advantageous given the invasiveness of CSF assessments and feasibility for long-term monitoring.

Based on a meta-analysis of > 60 studies, cognitive impairment, including overt dementia, has been linked to an increased risk of all-cause mortality [13]. Despite this evidence, the relationship between mortality and blood assay predictors for dementia, including plasma NfL, remains largely unexplored, particularly among dementia-free middle-aged adults. A few studies have examined plasma NfL and found associations with mortality in patients with stroke [14, 15], sporadic Creutzfeldt-Jakob disease [16], and spontaneous subarachnoid and intracerebral hemorrhages [17, 18] and thus focused on prognostic outcomes for specific clinical diagnoses. More recently, attention was drawn as to the possible prognostic value of plasma NfL in the general population. For instance, a study of elderly adults in the Memory and Morbidity in Augsburg Elderly (MEMO) study found that serum NfL levels were associated with all-cause mortality as well as neuropsychological test and brain atrophy scores [19]. Moreover, Kaeser et al. reported increasing plasma NfL levels with age in humans (*n* = 122; 21–107 years of age) and that plasma NfL correlated with plasma proteins involved in neural pathways. Importantly, they detected a positive association between plasma NfL levels and mortality among centenarians (*n* = 135) with a predictive value that exceeded that of cognitive and physical functioning measures, and this finding was replicated among nonagenarians in an independent cohort (*n* = 180) [20]. These data indicate that plasma NfL may be a novel blood-based biomarker of mortality in various neurological diseases but may also be an indicator of neuronal damage in non-demented adults.

In general, men consistently experience a lower survival probability at each age compared to women [21]. Thus, potential associations between plasma NfL and mortality should be tested both overall and separately among men and women. Moreover, the cardio-metabolic risk may also play a mediating and/or interactive role in this association, as they have been recently linked with elevated plasma NfL over time [22, 23, 24]. We therefore hypothesize that NfL and change in NfL over time interact with baseline cardio-metabolic conditions to affect mortality risk, while cardio-metabolic conditions’ effect on mortality risk may be mediated through NfL or change in NfL over time. A common framework examining both mediation and interaction can help test those associations simultaneously.

The present study examines the association between plasma NfL and all-cause mortality in a socio-economically diverse sample of middle-aged urban White and African American adults, overall and by sex. As a secondary objective, the study also tests the potential interactive and mediating effects of the body mass index (BMI), the allostatic load (AL) index, and other measures of cardio-metabolic risk on this relationship.

## Methods

### Study sample

Participants in this study were chosen from the Healthy Neighborhoods of Diversity Across the Life Span study [25]. HANDLS is an ongoing, prospective study of African American and White adults living in Baltimore, MD. Data from participants were collected from visit 1 (v_1_) from 2004 to 2009, visit 2 (v_2_) from 2009 to 2013, and visit 3 (v_3_) from 2013 to 2017. Each visit consisted of physical examinations, cognitive testing, and collection of fasting blood samples. Participants provided written informed consent.

Up to three repeats of plasma NfL concentrations were available from v_1_, v_2_, and/or v_3_. Among the 3720 initially recruited HANDLS participants, *N* = 731 had complete v_1_, v_2_, and/or v_3_ data of plasma NfL, of whom 694 had data on v_1_ plasma NfL (Additional file 1: Fig. S1). Given that all other covariates were either complete or imputed, the final analytic sample was *N* = 694 participants. The sample selection methodology for NfL is detailed in Additional file 1: Method S1 [26–29]. Compared to the unselected group (*N* = 3026 of 3720), the final eligible sample (*N* = 694) had a significantly higher proportion of White adults (44.2 vs. 40.1, *p* = 0.048) and of individuals with household incomes above poverty (70.9% vs. 56.0, *p* < 0.001), with no significant age or sex differences.

### Ethics

The HANDLS study is approved by the Institutional Review Board of the National Institutes of Health, National Institute of Environmental Health Sciences.

### Measures

#### Plasma neurofilament light measurement

Fasting blood samples were collected between 9:30 am and 11:30 am into EDTA blood collection tubes. The tubes were centrifuged at 600*g* for 15 min followed by the removal of the buffy coat. The steps were repeated twice and visually inspected for hemolysis. Plasma samples were aliquoted and stored at − 80 °C. Plasma NfL levels were quantified using the Simoa® NF-light Advantage Kit on a Simoa® HD-X analyzer by Quanterix (Billerica, MA, USA) following the kit instructions. Samples from the different visits were run on the same plate for each individual, and plates were balanced for individuals within each demographic group (race/sex/poverty). Plasma samples were diluted fourfold, and concentrations were adjusted for this dilution correction. Pooled plasma samples from two individuals were run in duplicate on all plates, and the average intra-assay and inter-assay coefficients of variations were 4.5% and 7%, respectively. Information on the limits of detection and quantification for this assay has previously been described [30]. Plasma NfL was measured with ≤ 3 repeats/participant, at v_1_, v_2_, and/or v_3_. The NfL_v1_ and δNfL exposures are detailed in Additional file 1: Method S2 [31]. NfL_v1_ is the baseline NfL measured at v_1_ and was Log_e_-transformed to approximate a normal distribution. Previously, we reported the δNfL as the annualized rate of change between NfLv_1_ and NfLv_2_ measurements [30]. Here, we included an additional measurement of NfL at v_3_. Therefore, in this study, the δNfL is the annualized rate of change between NfL_v1_, NfL_v2_, and NfLv_3_, on average, when these measurements were Log_e_-transformed.

#### Mortality status

Mortality status in the HANDLS cohort was obtained through linkages to the National Death Index (NDI), National Center for Health Statistics. Information about the underlying cause of death was obtained from death certificates and classified in accordance with the International Statistical Classification of Diseases, version 10 (ICD-10). Deaths attributed to CVD included CVD-related diagnosis codes (ICD-10 codes I00–99.9) listed as the underlying or contributing cause of death, respectively defined by the US Department of Health and Human Services and the Centers for Disease Control and Prevention as “the disease or injury that initiated the chain of morbid events that led directly and inevitably to death” and “all other significant diseases, conditions, or injuries that contributed to death but which did not result in the underlying cause of death” [32]. Vital status information for all participants is available from enrollment (2004–2009) to December 31, 2018 (last date of death available). However, in order to exclude participants who did not survive between visits 1 and 3, all death events occurred after the completion of the v_3_ exam (2013–2018) (Additional file 1: Method S1).

#### BMI, AL, and co-morbidity

Visit 1 BMI was calculated as weight (kg) divided by height (m^2^). We relied on a previously reported method to compute the total AL score [33], with components also measured at visit 1. This method sums cardiovascular (systolic and diastolic blood pressure, pulse rate), metabolic (total cholesterol, high-density lipoprotein (HDL) cholesterol, glycosylated Hb (HbA1C), sex-specific waist-to-hip ratio), and inflammatory (serum albumin and high-sensitivity C-reactive protein (hsCRP)) risk indicators, as summarized in Additional file 1: Table S1 [33–41]. The total AL score (AL_total_) is equally weighted and may range from 0 to 9. The higher the AL_total_, the more the overall cardio-metabolic risk. Total cholesterol (mg/dl), HDL cholesterol (mg/dl), hsCRP (mg/dl), albumin (g/dl), and glycosylated hemoglobin (%) were quantified by contract laboratories (Quest Diagnostics, Chantilly, VA), using reference analytical methods. Using standard protocols, trained examiners measured waist-to-hip ratio, radial pulse (beats/min), and systolic and diastolic blood pressure (mmHg). Specifically, blood pressure was measured using a mercury sphygmomanometer, and the arithmetic mean of left and right systolic and diastolic pressures was used in this analysis (Additional file 1: Table S2).

Cardio-metabolic and co-morbidity were assessed using self-reported, and measured components were assessed at visit 1. These included hypertension (0 = no, 1 = yes), diabetes (0 = non-diabetic, 1 = pre-diabetic, 2 = diabetic), dyslipidemia (or statin use) (0 = no, 1 = yes), and self-reported history of any of several cardiovascular disease conditions (0 = no, 1 = yes). The latter component screened for the occurrence of several conditions, namely atrial fibrillation, angina, coronary artery disease, congestive heart failure, and myocardial infarction.

BMI, AL (total score and parameters from which components were derived), and cardio-metabolic co-morbidity binary indices were considered both a potential mediator and an effect modifier in the association between NfL exposures and mortality.

#### Covariates

We assessed multiple other visit 1 covariates as potential confounders, given previous significant associations with plasma NfL and are considered antecedent risk factors to AL and cardio-metabolic risk. These included v_1_ age (continuous, years), sex (male, female), race (White, African American), poverty status (below vs. above 125% of the federal poverty line), and educational attainment (less than high school, high school, more than high school). We operationalized poverty status using the 2004 US Census Bureau poverty thresholds [42] based on household income and total family size (including children < 18 years). Few lifestyle and health-related factors were considered as potential confounders, namely current smoking status (0 = no vs. 1 = yes), illicit drug use (0 = no vs. 1 = yes, using any of marijuana, opiates, and cocaine), and the Healthy Eating Index 2010 (HEI-2010) [43], whereby overall diet quality was measured based on food and macronutrient-related dietary guidelines for Americans, total energy intake based on the average of two 24-h recalls (kcal/day), and the 20-item CES-D total score for depressive symptoms. Sex was the main effect modifier in our analyses.

### Analysis

We used Stata release 16 [44] to conduct all analyses. Data aside from outcome and exposures was imputed, using chained equations (5 imputations, 10 iterations) [45, 46], with most covariates having < 10% missing data compared to the final eligible sample (i.e., *N* = 694). We characterized the overall analytic sample at baseline using means and proportions; Student’s *t*-tests were used to examine the sex differences in baseline characteristics. We used a series of bivariate and multivariable regression models to evaluate whether baseline characteristics varied by sex. To examine the association between plasma NfL exposures, we estimated a series of Cox proportional hazard regression models with sequential covariate adjustment. Age (years) at the visit was used as the underlying time scale, while v_1_ age was the time of entry (i.e., delayed entry). Sex-specific Kaplan-Meier survival curves were presented across binary NfL exposures (> vs. ≤ median) based on the distributions in the final selected sample with a cutoff of 1.966279 for NfL_v1_ and 0.0466214 for δNfL on the Log_e_-transformed scale, examining the time on study as the analytic time variable. In Cox proportional hazards models, heterogeneity by sex of the association between NfL exposures and mortality was tested by adding a two-interaction term (NfL_v1_ × sex; δNfL × sex) in separate models. These models were also stratified by sex. The general modeling strategy consisted of a basic model, adjusted for age, sex, race, and poverty status (model 1), to which other lifestyle and health-related covariates (listed in the “Covariates” section) were subsequently added (model 2).

BMI, AL (total score and components), and cardio-metabolic co-morbidity were separately assessed as mediating/interactive factors in the total effect of NfL exposures on all-cause mortality. Continuous potential mediators (e.g., BMI, AL total score) were transformed into a standardized *z*-score, while binary cardio-metabolic co-morbidity indices were coded as 0 = no and 1 = yes/any, for ease of interpretation. Diabetes was recoded as 0 = no and 1 = pre-diabetic or diabetic. Continuous AL components were also tested individually as potential mediators/effect modifiers. Specifically, the overall effect of each of our main exposures on all-cause mortality, in the presence of a mediator with which the exposure may interact, was decomposed into four distinctive components: (i) neither mediation nor interaction, (ii) interaction alone (and not mediation), (iii) both mediation and interaction, and (iv) only mediation (but not interaction). This four-way decomposition unifies the methods to attribute effects to interactions and methods that assess mediation, and this method has recently been introduced in Stata, allowing to estimate four-way decomposition using parametric or semi-parametric regression models. Importantly, the *Med4way* command [47] [https://github.com/anddis/med4way] was used to test the mediation and interaction of the total effects of NfL exposure on mortality with several mediators/effect modifiers, using Cox PH models for the outcome and linear or logistic regression models for each mediator/effect modifier (BMI, AL total score, continuous AL components, and binary cardio-metabolic co-morbidity indices). Four-way decomposition was applied to the total sample, and among men and women, separately, combining the findings from 5 imputations using Rubin’s rule [48]. A logit link was specified for the mediating variable equation when mediators were binary. In a sensitivity analysis, NfL exposures (NfL_v1_ and δNfL) were entered as potential mediators while BMI, AL, AL continuous parameters, and co-morbidity indices were considered as the main exposure to assess the bidirectionality of the associations. In this analysis, all mediator equations were linear regression models. Total effects were interpreted as hazard ratios on the Log_e_ scale based on Cox PH models, per SD of exposures if the exposure was continuous and for “exposed” vs. “unexposed” if the exposure was binary, which were then decomposed into four components. An effect size that would result in a hazard ratio > 1.5 was considered as moderate to strong.

In all models, sample selectivity due to missing exposure and outcome data, relative to the initially recruited sample, was adjusted for using a two-stage Heckman selection strategy [49]. Thus, we first predicted an indicator of selection with socio-demographic factors, namely, v_1_ age, race, sex, and poverty status using a probit regression model, which yielded an inverse Mills ratio (IMR), a function of the probability of being selected given those socio-demographic factors. At a second stage, we estimated our Cox PH hazards regression models adjusted for the IMR in addition to the aforementioned covariates [30, 49]. A sensitivity analysis was also conducted to examine the sample selectivity by co-morbid conditions, while adjusting for socio-demographic factors, comparing the final analytic sample to those excluded from the original sample of *N* = 3720. We also compared the sex differences in co-morbidity in the final sample and the sample that was excluded.

## Results

Overall, 694 HANDLS participants were included in this study (Additional file 1: Fig. S1) and 42% were men and 55.8% were African American. The mean age at v_1_ was 47.8 years (30–65 years). After a mean follow-up of 11.2 years (range 3.86–14.31), 43 all-cause deaths occurred in the 694 participants (20 deaths among women, 23 deaths among men). The mean follow-up between v_1_ and v_3_ for δNfL was 7.77 years (range 4.9–12.5), when NfL was available for all 3 waves (*N* = 682). Overall, the incidence rate for all-cause death in the final eligible sample (*N* = 694) was 555 per 100,000 person-years (P-Y) (447 per 100,000 P-Y in women; 702 per 100,000 P-Y in men) and a median survival age of 78.1 years. Table 1 displays the sample characteristic distribution by sex, including the main exposures, covariates, potential mediators/effect modifiers, and the main outcome of interest. Most notably, men had significantly higher plasma NfL (Log_e_-transformed) compared to women at both visits 1 and 3 (*p* < 0.05). These differences remained comparable after further adjustment for age, race, and poverty status. Moreover, men were more likely than women to be pre-diabetic, with the reverse being observed in the case of diabetes. Self-reported history of CVD was more prevalent in women (15.9% vs. 8.7%, *p* = 0.007). While there was no sex difference detected in the mean AL_total_, several continuous components of AL were higher among women, namely hsCRP, total cholesterol, and HDL cholesterol, while the reverse was observed for albumin and DBP. Similar patterns were observed for binary AL components. Moreover, women had higher mean BMI compared to men, while having lower total caloric intakes and lower prevalence of current illicit drug use (*p* < 0.001). In a sensitivity analysis comparing those participants who were included in the analysis to those who were excluded, it was shown that inclusion was associated with a lower likelihood of diabetes, hypertension, and cardiovascular disease (*p* < 0.05). Moreover, among those excluded from the study, men were more likely than women to be pre-diabetic, as observed among those who were included. However, no sex differences were detected with respect to cardiovascular disease (*p* = 0.78), a finding that differed from the sex difference detected in the selected sample whereby men had a lower risk of CVD compared with women.

**Table 1:** Study sample characteristics by sex: HANDLS, 2004–2018^a^

	Overall (*N* = 694)	Women (*N* = 401)	Men (*N* = 293)	*P*_sex_
**Socio-demographic, lifestyle, and health-related factors at v**_**1**_				
% men	42.0	–	–	–
% African American	55.8	54.6	57.3	0.48
Age at v_1_, years	47.75 ± 0.34	47.94 ± 0.47	47.5 ± 0.5	0.52
% below poverty	29.1	30.9	26.6	0.22
Education %				
< High school	5.5	5.3	5.7	0.91
High school	57.1	56.7	57.8	–
> High school	37.4	38.0	36.5	0.71
Current illicit drug use, % yes	16.7	10.8	24.8	< 0.001^e^
Current tobacco use, % yes	40.1	40.0	47.2	0.065^e^
Healthy Eating Index, HEI-2010 total score	42.3 ± 0.5	42.8 ± 0.6	41.6 ± 0.8	0.26
Energy intake, kcal/day	1999 ± 46	1709 ± 49	2394 ± 71	< 0.001^e^
CES-D total score	14.3 ± 0.4	14.9 ± 0.6	13.5 ± 0.6	0.11
**Body mass index at v1, BMI**_**v1**_**, kg.m**^**-2**^	30.3 ± 0.3	31.8 ± 0.4	28.2 ± 0.4	< 0.001^e^
**Allostatic load total score at v**_**1**_**, AL**_**total**_	1.83 ± 0.04	1.86 ± 0.06	1.77 ± 0.07	0.36
**Continuous components**^**b**^**of the allostatic load at v**_**1**_**, AL**_**comp_cont**_				
Waist-to-hip ratio, WHR	0.944 ± 0.019	0.935 ± 0.032	0.955 ± 0.005	0.60
Serum albumin, g/dl	4.32 ± 0.01	4.27 ± 0.01	4.39 ± 0.02	< 0.001^e^
hsCRP, mg/L, Log_e_ transformed	0.738 ± 0.050	0.982 ± 0.064	0.401 ± 0.08	< 0.001^e^
Glycated hemoglobin, HbA1C, %	5.84 ± 0.04	5.84 ± 0.05	5.84 ± 0.06	1.00
Total cholesterol, mg/dl	186.6 ± 1.6	190.1 ± 2.03	181.8 ± 2.40	0.008^e^
HDL cholesterol, mg/dl	53.3 ± 0.6	55.8 ± 0.8	49.7 ± 1.0	< 0.001^e^
Resting heart rate, beat/min	66.8 ± 0.5	67.3 ± 0.6	66.2 ± 0.7	0.26
Systolic blood pressure, mmHg	119.1 ± 0.6	118.9 ± 0.9	119.4 ± 0.9	0.71
Diastolic blood pressure, mmHg	72.8 ± 0.4	71.9 ± 0.5	74.2 ± 0.6	0.003^e^
**Binary components**^**b**^**of the allostatic load at v**_**1**_**, AL**_**comp_bin**_				
Waist-to-hip ratio, WHR	76.8	77.4	76.0	0.69
Serum albumin, g/dl	2.2	3.0	1.0	0.093
hsCRP, mg/L	41.7	50.5	29.6	< 0.001^e^
Glycated hemoglobin, HbA1C, %	13.0	12.7	13.3	0.82
Total cholesterol, mg/dl	9.4	9.7	8.9	0.70
HDL cholesterol, mg/dl	19.0	13.0	27.0	< 0.001^e^
Resting heart rate, beat/min	3.1	3.0	3.2	0.87
Systolic blood pressure, mmHg	11.2	12.0	10.1	0.43
Diastolic blood pressure, mmHg	6.5	4.8	8.7	0.045^e^
**Co-morbidity**^c^				
Diabetes status, %				
No	68.5	71.5	64.5	–
Pre-diabetes	20.1	16.6	25.0	0.011^e^
Diabetes	11.3	11.9	10.5	0.069
Hypertension, %	40.9	43.6	37.3	0.10
Dyslipidemia, %	24.9	25.3	24.4	0.81
Cardiovascular disease, %	12.8	15.9	8.7	0.007^e^
**Plasma NfL, Log**_**e**_**transformed**				
NfL_v1_	1.981 ± 0.019	1.948 ± 0.024	2.026 ± 0.031	0.045^e^
NfL_v2_	2.179 ± 0.022	2.131 ± 0.027	2.245 ± 0.035	0.009^e^
NfL_v3_	2.348 ± 0.022	2.298 ± 0.028	2.417 ± 0.036	0.008^e^
δNfL^d^	0.0479 ± 0.0004	0.0472 ± 0.0005	0.0487 ± 0.0007	0.083^e^
**All-cause deaths, %**	6.20	5.00	7.8	0.13

*Abbreviations*: *AL*_*comp*_ allostatic load continuous components, *AL*_*total*_ allostatic load total score, *BMI* body mass index, *Bayes* empirical Bayes estimator, *CES-D* Center for Epidemiologic Studies-Depression, *δ* annualized rate of change, *HANDLS* Healthy Aging in Neighborhoods of Diversity Across the Life Span, *HDL* high-density lipoprotein, *HEI* Healthy Eating Index, *hsCRP* high-sensitivity C-reactive protein, *Hg* mercury, *NfL* plasma neurofilament light, *v*_*1*_ visit 1, *v*_*2*_ visit 2, *v*_*3*_ visit 3, *WHR* waist-to-hip ratio

^a^Values are means ± SE for continuous variables or % for categorical variables. SD for continuous variables can be computed as SE × sqrt(*N*)

^b^Continuous components of the AL were multiple imputed using chained equations. Binary outcomes were computed after imputation. See cutoffs for each component in supplemental methods

^c^Co-morbidity components were multiple imputed using chained equations. See definitions of components of co-morbidity in the “Methods” section

^d^Annual rate of change in NfL between v_1_ and v_3_ using the empirical Bayes estimator predicted from a mixed-effects linear regression model with NfL as the outcome and *TIME* as the only predictor, validated against the observed annualized change between v_1_ and v_3_ (Pearson’s *r* > 0.80)

^e^*p* < 0.05 upon further adjustment for age, race, and poverty status in multiple linear and multinomial logit models

**Fig. 1:**
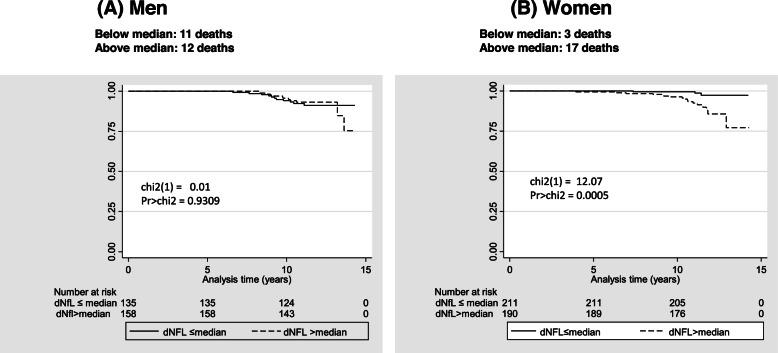
δNfL (above vs. below median) and all-cause mortality by sex: Kaplan-Meier survival curve (**a**, **b**). **a** dNfL corresponds to empirical Bayes estimator for change in Log_e_-transformed plasma NfL over v1, v2, and/or v3 of HANDLS (δNfL_bayes_ in Table 1). Below-median dNfL ranged between 0.0016891 and 0.0466214 with a mean ± SD of 0.0398147 ± 0.0055446; above-median dNfL ranged between 0.0466543 and 0.1086501, with a mean ± SD of 0.0559002 ± 0.0092975. Binary NfLv1 and δNfL (above vs. below or = median) based on the distributions in the final selected sample with a cutoff of 1.966279 for NfL_v1_ and 0.0466214 for δNfL. **b** Analyses were based on 401 women and 293 men selected at baseline of HANDLS with complete data on NfL exposures over 3 visits (see Method S1 for details). All-cause deaths through December 31, 2018: 20 deaths among women and 23 deaths among men. Analysis time is presented in years from v_1_ age

Figure 1 displays the findings from the Kaplan-Meier survival curve analyses across levels of δNfL (> vs. ≤ median: see Fig. 1 footnote for definitions), stratified by sex. The results show that only among women there was a marked difference in survivorship probability over time across those two levels, with log-rank test (df of 1) = 12.07, *p* = 0.0005, suggesting higher all-cause mortality risk in “> median” δNfL group vs. “≤ median.” Cox proportional hazards model and minimally and fully adjusted results are shown in Table 2, for both NfL_v1_ and δNfL, stratified by sex and expressed both as *z*-scores and binary exposures (above vs. below median). Similar to the findings from Fig. 1, binary δNfL among women was associated with increased risk for all-cause mortality in both the minimally adjusted model 1 (HR = 3.91, 95% CI 1.10–13.9, *p* = 0.036) and the fully adjusted model 2 (HR = 4.92, 95% CI 1.26–19.2, *p* = 0.022). When expressed as a standardized *z*-score, 1 SD increase in δNfL was associated with an increased risk of all-cause mortality among women, particularly in the fully adjusted model (HR = 1.65, 95% CI 1.04–2.61, *p* = 0.034). In both models 1 and 2, and among women, 1 SD of NfL_v1_ was associated with an increased risk of all-cause mortality (model 1: HR = 2.01, 95% CI 1.24–3.25, *p* = 0.005; model 2: HR = 1.75, 95% CI 1.02–2.98, *p* = 0.041), a relationship not detected for the binary NfL_v1_ exposure. Men did not exhibit a relationship between NfL exposure and mortality, and sex differences were detected for *z*-scored NfL_v1_ (model 1) and binary δNfL (both models).

**Table 2:** Plasma NfL_v1_ and δNfL and their relation to all-cause mortality by sex: Cox PH hazards models

	Overall (*N* = 694), *β* ± SE^a^	Women (*N* = 401), *β* ± SE	Men (*N* = 293), *β* ± SE	*P*_sex_^b^
**NfLv1**				
**Log**_**e**_**transformed,*z*-score**				
Model 1^d^	+ 0.275 ± 0.157, *p* = 0.080	+ 0.696 ± 0.247, *p* = 0.005	+ 0.060 ± 0.236, *p* = 0.80	0.018
	HR = 1.317	HR = 2.006	HR = 1.062	
Model 2^d^	+ 0.241 ± 0.170, *p* = 0.16	+ 0.557 ± 0.273, *p* = 0.041	+ 0.072 ± 0.257, *p* = 0.78	0.07
	HR = 1.273	HR = 1.745	HR = 1.075	
**Above vs. below median**^**e**^				
Model 1^d^	+ 0.177 ± 0.352, *p* = 0.61	+ 0.477 ± 0.527, *p* = 0.36	+ 0.104 ± 0.486, *p* = 0.83	0.34
	HR = 1.194	HR = 1.611	HR = 1.110	
Model 2^d^	0.095 ± 0.359, *p* = 0.79	+ 0.274 ± 0.566, *p* = 0.63	− 0.041 ± 0.509, *p* = 0.94	0.43
	HR = 1.010	HR = 1.315	HR = 0.956	
**δNfL**^c^				
**Log**_**e**_**transformed,*z*-score**				
Model 1^d^	+ 0.223 ± 0.141, *p* = 0.11	+ 0.436 ± 0.223, *p* = 0.051	+ 0.060 ± 0.197, *p* = 0.76	0.11
	HR = 1.250	HR = 1.547	HR = 1.062	
Model 2^d^	+ 0.201 ± 0.144, *p* = 0.16	+ 0.500 ± 0.235, *p* = 0.034	+ 0.047 ± 0.200, *p* = 0.81	0.092
	HR = 1.223	HR = 1.649	HR = 1.048	
**Above vs. below median**^**e**^				
Model 1^d^	+ 0.448 ± 0.346, *p* = 0.20	+ 1.363 ± 0.649, *p* = 0.036	− 0.194 ± 0.451, *p* = 0.67	0.015
	HR = 1.565	HR = 3.908	HR = 1.214	
Model 2^d^	+ 0.440 ± 0.351, *p* = 0.21	+ 1.595 ± 0.695, *p* = 0.022	− 0.266 ± 0.464, *p* = 0.57	0.011
	HR = 1.553	HR = 4.928	HR = 1.305	

*Abbreviations*: *Bayes* empirical Bayes estimator, *CES-D* Center for Epidemiologic Studies-Depression, *δ* annualized rate of change, *HANDLS* Healthy Aging in Neighborhoods of Diversity Across the Life Span, *HEI* Healthy Eating Index, *HR* hazard ratio, *NfL* plasma neurofilament light, *v*_*1*_ visit 1, *v*_*2*_ visit 2, *v*_*3*_ visit 3

^a^Values are Log_e_(HR) ± SE, *p* from Cox PH hazards models associated with each NfL exposure of interest. Hazard ratio (HR) point estimates are also presented. 95% CI for HR can be calculated as follows: lower confidence limit (LCL): exp[Log_e_HR − 1.96×SE(Log_e_HR)]; upper confidence limit (UCL): exp[Log_e_HR + 1.96×SE(Log_e_HR)]

^b^*P*-value for the 2-way interaction term between plasma NfL exposure and sex in a separate unstratified model

^c^Annual rate of change in NfL between v_1_ and v_3_ using the empirical Bayes estimator predicted from a mixed-effects linear regression model with NfL as the outcome and *TIME* as the only predictor, validated against the observed annualized change between v_1_ and v_3_ (Pearson’s *r* > 0.80)

^d^Model 1 adjusted for age at v1, sex, race, poverty status, and the inverse mills ratio; model 2 additionally adjusted for education, HEI-2010 total score, mean energy intake (kcal/day), current tobacco use, current illicit drug use, and the CES-D total score

^e^SD values of continuous exposures can be derived from Table 1. Binary NfLv1 and δNfL (above vs. below or = median) based on distributions in the final selected sample with a cutoff of 1.966279 for NfL_v1_ and 0.0466214 for δNfL

Tables 3 and 4 show the findings from 4-way decomposition models (secondary analyses), which decompose the total effect (TE) of the specified NfL exposure on all-cause mortality into components attributed to mediation alone (pure indirect effect [PIE]), interaction alone (reference interaction [IR]), to both mediation and interaction (mediated Interaction [IM]), and neither mediation nor interaction (controlled direct effect [CDE]). Many of the total effects observed among women were either non-significant, and most significant associations (*p* < 0.05) were direct ones that were not explained by or interacted with cardio-metabolic risk factors. Nevertheless, there was some evidence of an antagonistic interaction between hsCRP and NfL, whereby the effect of NfL at v_1_ was stronger at lower levels of hsCRP among women and while the controlled direct effect was stronger than the total effect (HR_cde,crp_ = 3.0, 95% CI 1.05–8.78, *p* = 0.04 vs. HR_te,crp_ = 2.3, 95% CI 0.73–7.21, *p* = 0.11).

**Table 3:** Plasma NfL_v1_ and its relation to all-cause mortality among women: mediating and interactive effects of BMI, AL, and co-morbidity index using 4-way decomposition^a,b,c^

	Overall (*N* = 694), *β* ± SE	*P*	Women (*N* = 401), *β* ± SE	*P*	Men (*N* = 293), *β* ± SE	*P*
***X*= NfL**_**v1**_**;*M*= BMI**						
Total effect	+ 0.397 ± 0.264	0.13	+ 0.877 ± 0.602	0.15	+ 0.138 ± 0.345	0.69
CDE	+ 0.173 ± 0.222	0.44	+ 0.712 ± 0.465	0.13	− 0.073 ± 0.228	0.75
IR	+ 0.083 ± 0.090	0.36	− 0.048 ± 0.191	0.80	+ 0.089 ± 0.198	0.65
IM	+ 0.106 ± 0.071	0.14	+ 0.276 ± 0.207	0.18	+ 0.031 ± 0.073	0.67
PM	+ 0.035 ± 0.050	0.48	− 0.064 ± 0.094	0.50	+ 0.090 ± 0.065	0.16
***X*= NfL**_**v1**_**;*M*= AL**						
Total effect	+ 0.283 ± 0.220	0.20	+ 0.835 ± 0.585	0.15	+ 0.120 ± 0.267	0.65
CDE	+ 0.282 ± 0.219	0.20	+ 0.857 ± 0.586	0.14	− 0.009 ± 0.253	0.97
IR	+ 0.000 ± 0.011	1.00	− 0.013 ± 0.037	0.72	+ 0.079 ± 0.115	0.49
IM	+ 0.004 ± 0.015	0.80	+ 0.004 ± 0.018	0.84	+ 0.033 ± 0.036	0.36
PM	− 0.003 ± 0.015	0.83	− 0.011 ± 0.021	0.59	+ 0.017 ± 0.032	0.61
***X*= NfL**_**v1**_**;*M*= WHR**						
Total effect	+ 0.540 ± 2.051	0.79	+ 0.592 ± 1.976	0.76	+ 0.097 ± 0.307	0.75
CDE	+ 0.246 ± 0.246	0.32	+ 0.609 ± 1.02	0.55	+ 0.068 ± 0.293	0.82
IR	+ 0.400 ± 2.391	0.87	+ 0.114 ± 1.672	0.95	+ 0.005 ± 0.059	0.93
IM	− 0.095 ± 0.360	0.79	− 0.051 ± 0.429	0.91	+ 0.008 ± 0.041	0.84
PM	− 0.012 ± 0.099	0.91	− 0.080 ± 0.294	0.79	+ 0.016 ± 0.030	0.61
***X*= NfL**_**v1**_**;*M*= ALB**						
Total effect	+ 0.239 ± 0.210	0.25	+ 0.728 ± 0.485	0.13	− 0.002 ± 0.242	1.00
CDE	+ 0.236 ± 0.214	0.27	+ 0.773 ± 0.484	0.11	− 0.007 ± 0.250	0.98
IR	− 0.003 ± 0.026	0.91	− 0.039 ± 0.069	0.58	− 0.041 ± 0.060	0.50
IM	+ 0.019 ± 0.022	0.42	− 0.010 ± 0.022	0.66	+ 0.046 ± 0.034	0.17
PM	− 0.011 ± 0.021	0.61	+ 0.003 ± 0.015	0.85	− 0.000 ± 0.049	0.99
***X*= NfL**_**v1**_**;*M*= CRP**						
Total effect	+ 0.376 ± 0.245	0.13	+ 0.829 ± 0.585	0.16	+ 0.132 ± 0.289	0.65
CDE	+ 0.399 ± 0.244	0.10	**+ 1.109 ± 0.543**	**0.041**	+ 0.045 ± 0.310	0.88
IR	− 0.017 ± 0.029	0.55	− 0.274 ± 0.162	0.091	+ 0.074 ± 0.110	0.50
IM	+ 0.015 ± 0.022	0.49	+ 0.064 ± 0.058	0.27	+ 0.025 ± 0.029	0.39
PM	− 0.021 ± 0.021	0.32	− 0.071 ± 0.053	0.18	− 0.013 ± 0.022	0.57
***X*= NfL**_**v1**_**;*M*= HBA1C**						
Total effect	+ 0.275 ± 0.222	0.22	+ 0.884 ± 0.565	0.12	+ 0.082 ± 0.277	0.77
CDE	+ 0.264 ± 0.224	0.24	+ 0.864 ± 0.564	0.13	+ 0.084 ± 0.278	0.76
IR	− 0.001 ± 0.012	0.94	− 0.007 ± 0.022	0.74	+ 0.001 ± 0.023	0.98
IM	+ 0.001 ± 0.008	0.93	+0.003 ± 0.015	0.82	− 0.005 ± 0.014	0.74
PM	+ 0.011 ± 0.014	0.43	+ 0.023 ± 0.024	0.34	+ 0.002 ± 0.016	0.89
***X*= NfL**_**v1**_**;*M*= CHOL**						
Total effect	+ 0.244 ± 0.220	0.27	+ 0.727 ± 0.478	0.13	− 0.002 ± 0.277	0.99
CDE	+ 0.247 ± 0.219	0.26	+ 0.715 ± 0.481	0.14	+ 0.043 ± 0.258	0.87
IR	− 0.000 ± 0.015	0.98	+ 0.017 ± 0.059	0.78	− 0.053 ± 0.071	0.46
IM	− 0.007 ± 0.012	0.59	− 0.002 ± 0.008	0.83	− 0.030 ± 0.038	0.43
PM	+ 0.004 ± 0.014	0.79	− 0.003 ± 0.012	0.81	+ 0.037 ± 0.041	0.36
***X*= NfL**_**v1**_**;*M*= HDL**						
Total effect	+ 0.436 ± 0.280	0.12	+ 1.166 ± 0.714	0.10	+ 0.106 ± 0.332	0.75
CDE	+ 0.298 ± 0.239	0.21	+ 0.672 ± 0.484	0.17	+ 0.083 ± 0.330	0.80
IR	+ 0.072 ± 0.072	0.32	+ 0.365 ± 0.311	0.24	− 0.007 ± 0.036	0.85
IM	+ 0.050 ± 0.038	0.20	+ 0.104 ± 0.098	0.29	+ 0.010 ± 0.060	0.87
PM	+ 0.016 ± 0.024	0.50	+ 0.026 ± 0.036	0.47	− 0.020 ± 0.041	0.63
***X*= NfL**_**v1**_**;*M*= RHR**						
Total effect	+ 0.310 ± 0.229	0.18	+ 0.725 ± 0.515	0.16	+ 0.215 ± 0.327	0.51
CDE	+ 0.348 ± 0.239	0.15	+ 0.757 ± 0.530	0.15	+ 0.228 ± 0.324	0.48
IR	− 0.029 ± 0.040	0.47	+ 0.020 ± 0.085	0.81	− 0.013 ± 0.113	0.91
IM	+ 0.004 ± 0.009	0.66	− 0.015 ± 0.025	0.55	+ 0.005 ± 0.029	0.87
PM	− 0.014 ± 0.016	0.39	− 0.037 ± 0.037	0.32	− 0.004 ± 0.020	0.84
***X*= NfL**_**v1**_**;*M*= SBP**						
Total effect	+ 0.247 ± 0.225	0.27	+ 0.759 ± 0.495	0.13	+ 0.046 ± 0.278	0.87
CDE	+ 0.257 ± 0.227	0.26	+ 0.769 ± 0.495	0.12	+ 0.044 ± 0.284	0.88
IR	+ 0.003 ± 0.012	0.83	− 0.000 ± 0.008	0.97	+ 0.015 ± 0.058	0.79
IM	− 0.006 ± 0.017	0.71	+ 0.001 ± 0.037	0.99	− 0.009 ± 0.021	0.67
PM	− 0.007 ± 0.021	0.76	− 0.010 ± 0.049	0.84	− 0.003 ± 0.019	0.88
***X*= NfL**_**v1**_**;*M*= DBP**						
Total effect	+ 0.268 ± 0.218	0.22	+ 0.812 ± 0.529	0.13	+ 0.093 ± 0.280	0.74
CDE	+ 0.291 ± 0.221	0.19	+ 0.912 ± 0.558	0.10	+ 0.111 ± 0.290	0.70
IR	− 0.002 ± 0.030	0.95	+ 0.029 ± 0.090	0.75	− 0.015 ± 0.072	0.83
IM	− 0.001 ± 0.021	0.97	− 0.090 ± 0.096	0.34	+ 0.001 ± 0.008	0.88
PM	− 0.020 ± 0.021	0.33	− 0.038 ± 0.057	0.51	− 0.004 ± 0.013	0.76
***X*= NfL**_**v1**_**;*M*= HYPERT**						
Total effect	+ 0.288 ± 0.217	0.19	+ 0.729 ± 0.489	0.14	+ 0.077 ± 0.271	0.78
CDE	+ 0.080 ± 0.232	0.73	+ 0.096 ± 0.347	0.78	+ 0.174 ± 0.354	0.62
IR	+ 0.199 ± 0.159	0.21	+ 0.600 ± 0.379	0.11	− 0.092 ± 0.167	0.58
IM	+ 0.006 ± 0.013	0.64	+ 0.020 ± 0.053	0.71	− 0.003 ± 0.010	0.76
PM	+ 0.003 ± 0.008	0.69	+ 0.013 ± 0.034	0.71	− 0.002 ± 0.008	0.81
***X*= NfL**_**v1**_**;*M*= DIAB**						
Total effect	+ 0.370 ± 0.244	0.13	+ 0.714 ± 0.485	0.14	+ 0.158 ± 0.314	0.62
CDE	+ 0.484 ± 0.295	0.10	+ 0.760 ± 0.501	0.13	+ 0.314 ± 0.431	0.47
IR	− 0.117 ± 0.120	0.32	+ 0.032 ± 0.225	0.89	− 0.174 ± 0.175	0.32
IM	+ 0.016 ± 0.019	0.38	− 0.007 ± 0.051	0.89	+ 0.012 ± 0.020	0.56
PM	− 0.014 ± 0.019	0.46	− 0.070 ± 0.057	0.21	+ 0.006 ± 0.014	0.68
***X*= NfL**_**v1**_**;*M*= HYPERCHOL**						
Total effect	+ 0.279 ± 0.244	0.25	+ 0.637 ± 0.481	0.19	+ 0.137 ± 0.338	0.69
CDE	+ 0.270 ± 0.300	0.37	+ 0.566 ± 0.528	0.28	+ 0.182 ± 0.414	0.66
IR	+ 0.006 ± 0.089	0.95	+ 0.078 ± 0.141	0.58	− 0.047 ± 0.091	0.61
IM	− 0.001 ± 0.006	0.83	− 0.010 ± 0.021	0.64	+ 0.000 ± 0.007	0.99
PM	+ 0.004 ± 0.009	0.67	+ 0.003 ± 0.018	0.87	+ 0.002 ± 0.020	0.94
***X*= NfL**_**v1**_**;*M*= CVD**						
Total effect	+ 0.158 ± 0.213	0.46	+ 0.362 ± 0.414	0.38	+ 0.055 ± 0.282	0.85
CDE	+ 0.078 ± 0.230	0.73	+ 0.260 ± 0.446	0.56	+ 0.007 ± 0.285	0.98
IR	+ 0.080 ± 0.053	0.13	+ 0.102 ± 0.122	0.41	+ 0.049 ± 0.057	0.40
IM	+ 0.002 ± 0.011	0.87	+ 0.002 ± 0.019	0.90	+ 0.000 ± 0.011	1.00
PM	− 0.002 ± 0.012	0.84	− 0.003 ± 0.024	0.90	− 0.000 ± 0.009	0.99

*Abbreviations*: *AL* allostatic load, *ALB* albumin, *BMI* body mass index, *CDE* controlled direct effect, *CES-D* Center for Epidemiological Studies-Depression, *CHOL* total cholesterol, *CRP* C-reactive protein (high sensitivity), Log_e_ transformed, *CVD* cardiovascular disease, *DBP* diastolic blood pressure, *DIAB* diabetes, *HBA1C* glycated hemoglobin, *HDL* high-density lipoprotein cholesterol, *HEI-2010* Healthy Eating Index-2010 version, *HYPERT* hypertension, *HYPERCHOL* hypercholesterolemia, *IM* interaction, mediated, *IR* interaction, reference, *M* mediators/effect modifier, *NfL* plasma neurofilament light chain, Log_e_ transformed, *PM* pure mediation, *RHR* resting heart rate, *SBP* systolic blood pressure, *WHR* waist-hip ratio, *X* exposure

^a^See the “Methods” section and Table 1 for the definition of each NfL exposure (i.e., NfLv1 and δNfL). All exposures (*X*) and potential mediators/effect modifiers (*M*) were *z*-scored for ease of interpretation, with the exception of binary *M* (coded as 0/1), namely DIAB, HYPERT, HYPERCHOL, and CVD. Control variables were set at their means

^b^Cox models for which 4-way decomposition was conducted are equivalent to model 2 (Table 2), for continuous exposures, to which *M* was added and considered as a potential mediator/effect modifier. Control variables included age at v1, sex, race, poverty status, education, HEI-2010 total score, mean energy intake (kcal/day), current tobacco use, current illicit drug use, the CES-D total score, and the inverse mills ratio

^c^Total effects are beta = Log_e_(HR) ± SE with associated *p*-values from Cox PH hazards models associated with each NfL exposure of interest. Hazard ratio (HR) point estimates the exponent of beta. 95% CI for HR can be calculated as follows: lower confidence limit (LCL): exp[Log_e_HR − 1.96×SE(Log_e_HR)]; upper confidence limit (UCL): exp[Log_e_HR + 1.96×SE(Log_e_HR)]

**Table 4:** Plasma δNfL and its relation to all-cause mortality, overall, and by sex: mediating and interactive effects of BMI, AL, and co-morbidity index using 4-way decomposition^a,b,c^

	Overall (*N* = 694), *β* ± SE	*P*	Women (*N* = 401), *β* ± SE	*P*	Men (*N* = 293), *β* ± SE	*P*
***X*= δNfL;*M*= BMI**						
Total effect	+ 0.201 ± 0.170	0.24	+ 0.680 ± 0.386	0.079	− 0.052 ± 0.208	0.80
CDE	+ 0.264 ± 0.171	0.12	+ 0.750 ± 0.417	0.072	+ 0.090 ± 0.173	0.60
IR	− 0.043 ± 0.044	0.33	− 0.025 ± 0.086	0.77	− 0.126 ± 0.138	0.36
IM	+ 0.015 ± 0.017	0.36	+ 0.016 ± 0.060	0.79	+ 0.011 ± 0.017	0.52
PM	− 0.036 ± 0.024	0.14	− 0.064 ± 0.058	0.27	− 0.027 ± 0.032	0.40
***X*= δNfL;*M*= AL**						
Total effect	+ 0.229 ± 0.179	0.20	+ 0.637 ± 0.389	0.10	+ 0.056 ± 0.228	0.81
CDE	+ 0.230 ± 0.182	0.20	+ 0.567 ± 0.405	0.15	+ 0.059 ± 0.213	0.78
IR	+ 0.001 ± 0.011	0.91	+ 0.011 ± 0.050	0.82	+ 0.015 ± 0.072	0.84
IM	− 0.002 ± 0.020	0.90	+ 0.019 ± 0.052	0.71	− 0.004 ± 0.021	0.84
PM	− 0.000 ± 0.018	0.99	+ 0.019 ± 0.047	0.68	− 0.014 ± 0.023	0.55
***X*= δNfL;*M*= WHR**						
Total effect	+ 0.866 ± 2.644	0.74	+ 1.33 ± 5.07	0.79	+ 0.096 ± 0.249	0.70
CDE	+ 0.198 ± 0.180	0.27	+ 0.55 ± 0.57	0.34	+ 0.090 ± 0.255	0.72
IR	+ 0.798 ± 2.986	0.79	+ 0.98 ± 6.12	0.87	+ 0.019 ± 0.065	0.77
IM	− 0.117 ± 0.353	0.74	− 0.18 ± 0.90	0.84	− 0.003 ± 0.032	0.92
PM	− 0.012 ± 0.074	0.87	− 0.02 ± 0.17	0.91	− 0.011 ± 0.020	0.60
***X*= δNfL;*M*= ALB**						
Total effect	+ 0.107 ± 0.167	0.52	+ 0.651 ± 0.382	0.088	− 0.170 ± 0.185	0.36
CDE	+ 0.090 ± 0.170	0.60	+ 0.605 ± 0.383	0.11	− 0.133 ± 0.189	0.48
IR	+ 0.021 ± 0.058	0.71	+ 0.047 ± 0.127	0.71	− 0.026 ± 0.122	0.83
IM	− 0.005 ± 0.014	0.69	− 0.001 ± 0.011	0.94	− 0.013 ± 0.029	0.66
PM	− 0.002 ± 0.005	0.75	− 0.000 ± 0.005	0.99	+ 0.002 ± 0.007	0.80
***X*= δNfL;*M*= CRP**						
Total effect	+ 0.164 ± 0.175	0.35	+ 0.578 ± 0.362	0.11	− 0.032 ± 0.217	0.88
CDE	+ 0.171 ± 0.174	0.33	+ 0.669 ± 0.371	0.072	− 0.036 ± 0.228	0.88
IR	− 0.008 ± 0.002	0.65	− 0.100 ± 0.107	0.35	+ 0.008 ± 0.038	0.84
IM	− 0.000 ± 0.003	0.88	− 0.024 ± 0.037	0.52	+ 0.003 ± 0.015	0.83
PM	+ 0.001 ± 0.006	0.87	+ 0.034 ± 0.037	0.35	− 0.007 ± 0.020	0.71
***X*= δNfL;*M*= HBA1C**						
Total effect	+ 0.079 ± 0.183	0.67	+ 0.393 ± 0.425	0.36	− 0.091 ± 0.222	0.68
CDE	+ 0.159 ± 0.155	0.31	+ 0.470 ± 0.370	0.20	+ 0.011 ± 0.191	0.95
IR	− 0.077 ± 0.095	0.42	− 0.058 ± 0.135	0.67	− 0.103 ± 0.145	0.48
IM	**+ 0.056 ± 0.023**	**0.014**	+ 0.042 ± 0.036	0.25	+ 0.061 ± 0.034	0.071
PM	− 0.059 ± 0.041	0.15	− 0.061 ± 0.080	0.44	+ 0.059 ± 0.050	0.23
***X*= δNfL;*M*= CHOL**						
Total effect	+ 0.312 ± 0.195	0.11	+ 0.682 ± 0.400	0.089	+ 0.116 ± 0.244	0.64
CDE	+ 0.303 ± 0.194	0.12	+ 0.535 ± 0.381	0.16	+ 0.168 ± 0.264	0.52
IR	− 0.003 ± 0.046	0.95	+ 0.101 ± 0.147	0.49	− 0.045 ± 0.064	0.48
IM	+ 0.023 ± 0.018	0.20	+ 0.042 ± 0.046	0.37	+ 0.011 ± 0.019	0.55
PM	− 0.012 ± 0.015	0.43	+ 0.004 ± 0.025	0.87	− 0.018 ± 0.022	0.42
***X*= δNfL;*M*= HDL**						
Total effect	+ 0.230 ± 0.182	0.21	+ 0.699 ± 0.411	0.089	+ 0.016 ± 0.216	0.94
CDE	+ 0.222 ± 0.171	0.20	+ 0.736 ± 0.402	0.067	− 0.000 ± 0.230	1.00
IR	+ 0.002 ± 0.037	0.95	− 0.024 ± 0.180	0.89	+ 0.014 ± 0.050	0.77
IM	+ 0.001 ± 0.004	0.85	− 0.001 ± 0.012	0.97	− 0.004 ± 0.011	0.72
PM	+ 0.005 ± 0.010	0.65	− 0.013 ± 0.029	0.66	+ 0.006 ± 0.012	0.64
***X*= δNfL;*M*= RHR**						
Total effect	+ 0.223 ± 0.171	0.20	+ 0.599 ± 0.382	0.12	+ 0.032 ± 0.191	0.87
CDE	+ 0.220 ± 0.172	0.20	+ 0.425 ± 0.385	0.27	+ 0.062 ± 0.189	0.74
IR	− 0.022 ± 0.032	0.50	+ 0.085 ± 0.124	0.50	− 0.031 ± 0.070	0.66
IM	− 0.012 ± 0.021	0.58	+ 0.051 ± 0.056	0.36	− 0.031 ± 0.029	0.29
PM	+ 0.036 ± 0.025	0.16	+ 0.037 ± 0.049	0.45	+ 0.032 ± 0.033	0.33
***X*= δNfL;*M*= SBP**						
Total effect	+ 0.211 ± 0.176	0.23	+ 0.654 ± 0.389	0.093	+ 0.009 ± 0.208	0.97
CDE	+ 0.190 ± 0.181	0.29	+ 0.639 ± 0.385	0.097	− 0.0236 ± 0.221	0.92
IR	+ 0.006 ± 0.019	0.76	+ 0.005 ± 0.039	0.89	+ 0.010 ± 0.039	0.80
IM	+ 0.010 ± 0.018	0.58	+ 0.001 ± 0.041	0.83	+ 0.020 ± 0.030	0.52
PM	+ 0.005 ± 0.019	0.79	+ 0.001 ± 0.025	0.98	+ 0.003 ± 0.038	0.94
***X*= δNfL;*M*= DBP**						
Total effect	+ 0.248 ± 0.180	0.17	+ 0.678 ± 0.404	0.094	+ 0.043 ± 0.207	0.84
CDE	+ 0.248 ± 0.184	0.18	+ 0.682 ± 0.398	0.087	− 0.020 ± 0.230	0.93
IR	− 0.010 ± 0.024	0.68	− 0.014 ± 0.042	0.74	+ 0.031 ± 0.086	0.72
IM	− 0.005 ± 0.014	0.74	− 0.002 ± 0.018	0.93	+ 0.015 ± 0.036	0.68
PM	+ 0.015 ± 0.017	0.36	+ 0.011 ± 0.020	0.58	+ 0.017 ± 0.036	0.63
***X*= δNfL;*M*= HYPERT**						
Total effect	+ 0.248 ± 0.185	0.18	+ 0.666 ± 0.392	0.089	+ 0.138 ± 0.259	0.59
CDE	+ 0.322 ± 0.261	0.22	+ 0.252 ± 0.369	0.50	+ 0.377 ± 0.416	0.37
IR	− 0.089 ± 0.140	0.53	+ 0.296 ± 0.338	0.38	− 0.181 ± 0.172	0.30
IM	− 0.016 ± 0.025	0.53	+ 0.047 ± 0.059	0.43	+ 0.043 ± 0.045	0.33
PM	+ 0.031 ± 0.028	0.28	+ 0.071 ± 0.051	0.16	− 0.015 ± 0.042	0.73
***X*= δNfL;*M*= DIAB**						
Total effect	+ 0.153 ± 0.178	0.39	+ 0.644 ± 0.395	0.10	− 0.072 ± 0.222	0.75
CDE	+ 0.037 ± 0.237	0.88	+ 0.206 ± 0.396	0.60	− 0.190 ± 0.336	0.57
IR	+ 0.098 ± 0.096	0.31	+ 0.321 ± 0.258	0.22	+ 0.123 ± 0.134	0.36
IM	+ 0.021 ± 0.021	0.33	+ 0.093 ± 0.084	0.27	+ 0.022 ± 0.026	0.41
PM	− 0.002 ± 0.023	0.94	+ 0.023 ± 0.057	0.69	− 0.026 ± 0.028	0.37
***X*= δNfL;*M*= HYPERCHOL**						
Total effect	+ 0.207 ± 0.178	0.25	+ 0.552 ± 0.380	0.15	+ 0.023 ± 0.209	0.91
CDE	+ 0.128 ± 0.207	0.54	+ 0.407 ± 0.440	0.36	+ 0.003 ± 0.252	0.99
IR	+ 0.081 ± 0.078	0.30	+ 0.136 ± 0.163	0.41	+ 0.023 ± 0.083	0.79
IM	+ 0.006 ± 0.011	0.56	+ 0.023 ± 0.033	0.49	+ 0.001 ± 0.006	0.92
PM	− 0.008 ± 0.012	0.49	− 0.014 ± 0.023	0.55	− 0.004 ± 0.021	0.86
***X*= δNfL;*M*= CVD**						
Total effect	+ 0.203 ± 0.172	0.24	+ 0.542 ± 0.385	0.16	+ 0.047 ± 0.211	0.82
CDE	+ 0.146 ± 0.184	0.43	+ 0.485 ± 0.434	0.26	+ 0.054 ± 0.228	0.81
IR	+ 0.057 ± 0.048	0.23	+ 0.060 ± 0.109	0.58	+ 0.002 ± 0.036	0.96
IM	+ 0.011 ± 0.012	0.34	+ 0.004 ± 0.013	0.75	+ 0.001 ± 0.019	0.95
PM	− 0.010 ± 0.012	0.38	− 0.009 ± 0.017	0.64	− 0.009 ± 0.023	0.68

*Abbreviations*: *AL* allostatic load, *ALB* albumin, *BMI* body mass index, *CDE* controlled direct effect, *CES-D* Center for Epidemiological Studies-Depression, *CHOL* total cholesterol, *CRP* C-reactive protein (high sensitivity), Log_e_ transformed, *CVD* cardiovascular disease, *DBP* diastolic blood pressure, *DIAB* diabetes, *HBA1C* glycated hemoglobin, *HDL* high-density lipoprotein cholesterol, *HEI-2010* Healthy Eating Index-2010 version, *HYPERT* hypertension, *HYPERCHOL* hypercholesterolemia, *IM* interaction, mediated, *IR* interaction, reference, *M* mediators/effect modifier, *NfL* plasma neurofilament light chain, Log_e_ transformed, *PM* pure mediation, *RHR* resting heart rate, *SBP* systolic blood pressure, *WHR* waist-hip ratio, *X* exposure

^a^See the “Methods” section and Table 1 for the definition of each NfL exposure (i.e., NfLv1 and δNfL). All exposures (*X*) and potential mediators/effect modifiers (*M*) were *z*-scored for ease of interpretation, with the exception of binary *M* (coded as 0/1), namely DIAB, HYPERT, HYPERCHOL, and CVD. Control variables were set at their means

^b^Cox models for which 4-way decomposition was conducted are equivalent to model 2 (Table 2), for continuous exposures, to which *M* was added and considered as a potential mediator/effect modifier. Control variables included age at v1, sex, race, poverty status, education, HEI-2010 total score, mean energy intake (kcal/day), current tobacco use, current illicit drug use, the CES-D total score, and the inverse mills ratio

^c^Total effects are beta = Log_e_(HR) ± SE with associated *p*-values from Cox PH hazards models associated with each NfL exposure of interest. Hazard ratio (HR) point estimates the exponent of beta. 95% CI for HR can be calculated as follows: lower confidence limit (LCL): exp[Log_e_HR − 1.96×SE(Log_e_HR)]; upper confidence limit (UCL) exp[Log_e_HR + 1.96×SE(Log_e_HR)]

Moreover, we also examined the potential effects of each cardio-metabolic risk and co-morbidity factors in relation to all-cause mortality by sex, exploring the mediating and modifying effects with NfL_v1_ and δNfL as the main exposures (Additional file 1: Tables S1 and S2). Based on the findings presented in Additional file 1: Table S2, among men, when NfL_v1_ is set at its mean, BMI becomes potentially protective against all-cause mortality (*β* ± SE − 0.441 ± 0.196, *p* = 0.024). A potential protective effect of CVD history on mortality would be observed among women, if NfL_v1_ is set at this mean (*β* ± SE − 0.803 ± 0.249, *p* = 0.001). When δNfL was tested as the mediating and interactive factor for the association between cardio-metabolic risk and co-morbidity vs. all-cause mortality, several notable findings were observed. First, among men, when δNfL is set at its mean, BMI has an inverse association with all-cause mortality. The marginally significant inverse relationship between HbA1c and all-cause mortality observed when δNfL is set at its mean (CDE < 0, *p* < 0.10) becomes non-significant when δNfL is not fixed in the total population with this difference in effect caused by pure interaction as well as mediated interaction (IR > 0, IM > 0, *p* < 0.05), indicative of synergism between HbA1c and δNfL in determining mortality risk. Finally, among men, a CDE of − 0.684 (*p* = 0.037) for hypercholesterolemia was found when δNfL was set at its mean, indicating that at an average rate of change in NfL over time, hypercholesterolemia is potentially protective against all-cause mortality.

## Discussion

Few studies have examined the association of plasma NfL and mortality in population-based studies, and these studies have been conducted in elderly White adults [19]. In this study, we examined the association between plasma NfL and all-cause mortality in a socio-economically diverse sample of middle-aged urban White and African American adults. Furthermore, we analyzed these associations both overall and by sex. In addition, it is also the first to test the potential interactive and mediating effects of BMI, AL index, and other measures of cardio-metabolic risk on this relationship. Here, we report that in women, δNfL was associated with an increased risk of all-cause mortality. In women, 1 SD of NfL_v1_ was associated with an increased risk of all-cause mortality, a relationship not detected for the binary NfL_v1_ exposure. Men did not exhibit a relationship between NfL exposure and mortality. We further explored these associations and found that in women, most of these associations were direct and were not explained by or did not interact with cardio-metabolic risk factors. We found one exception with a possible antagonistic interaction for hsCRP and NfL_v1_, indicating that NfL_v1_ is a better prognostic indicator at normal hsCRP values. Moreover, there was some evidence of synergistic interaction between HbA1c and δNfL in determining mortality risk, overall. These novel results add informative insight that plasma NfL can potentially be used as a biomarker to predict all-cause mortality in middle-aged women across different races.

Plasma NfL has recently gained significant attention due to its association with neurological diseases including sporadic and familial AD [5, 7, 50], frontotemporal degeneration [10], multiple sclerosis [12], traumatic brain injury [11], Parkinson’s disease [4], and other neurological disorders [9]. In addition, levels of plasma NfL predict the future onset of dementia [8, 51]. In non-demented adults, plasma NfL may also have clinical utility as it has been shown to be associated with a faster decline in normalized mental status scores in White adults and in older adults [30]. In older adults (median age 75 years) in the Multidomain Alzheimer’s Preventive Trial (MAPT), plasma NfL was associated with cognitive scores and executive function in adults with MCI but not in adults without cognitive impairment [52]. Blood-based biomarkers can be powerful tools for assessing the risk and diagnosing of disease and have the advantage of being easier to obtain, less expensive, and not requiring highly specialized clinical staff as well as equipment that is typically needed for the acquisition of CSF and neuroimaging modalities.

As more and more studies test the predictive value of plasma NfL, we develop a clearer understanding of what blood levels of this biomarker may indicate. Yet, we still do not fully understand what a rise in plasma NfL levels may signify in the absence of a disease diagnosis. To gain further insight, recent studies have examined the plasma NfL levels and their association with mortality. Higher plasma NfL levels are associated with mortality in patients with stroke [14, 15], sporadic Creutzfeldt-Jakob disease [16], and spontaneous subarachnoid and intracerebral hemorrhages [17, 18]. In these patient cohorts, brain injury initiates neuroaxonal damage which would lead to the release of neuronal NfL into the peripheral blood [9]. Higher NfL in these cases of acute neuroaxonal damage predicts short-term mortality as most of these studies had ~ 30-day follow-up periods, with the exception of sporadic Creutzfeldt-Jakob disease which had a longer follow-up period (mean 14.8 months) [16]. In these instances of acute brain injury, plasma NfL has the utility to predict short-term mortality. However, little evidence exists as to whether NfL is associated with future risk of all-cause mortality in the general population. A recent report found that plasma NfL was associated with mortality in centenarians and nonagenarians, with no reported sex differences in these associations or in plasma NfL levels [20]. Data from the MEMO study of elderly adults (*N* = 386) found that serum NfL levels were associated with all-cause mortality [19]. In this study, the adjusted hazard ratio obtained from Cox proportional hazards analysis was stronger among men compared with that among women. This is in contrast to our study where significant associations between plasma NfL and mortality were only observed in women. These differences may be multi-factorial in that samples from the participants in the MEMO study were from ~ 1997 and the participants were White and leaner (mean BMI = 27.7) with a mean age of 73 years living in Augsburg, Germany. Our study consisted of African American and White adults with a mean age of 47.75 and a higher BMI (mean = 30.3). Nevertheless, in these population-based studies, blood-based NfL was associated with mortality in women. These studies shed light that a blood assay predictor of dementia is also associated with all-cause mortality in the absence of neurological disease. This suggests that plasma/serum NfL may be used as a clinical biomarker to identify individuals at high mortality risk.

Moreover, a recent analysis of the same cohort as this current study, examined the associations of BMI, allostatic load index (AL_index_ and its continuous components) with change in plasma NfL over time. This analysis indicated that HbA1c plays an important role in mediating the associations of BMI and the AL_index_ with δNfL [24]. This partly explains our finding that HbA1c may interact with change in NfL over time to determine mortality risk. Another finding with continuous AL components indicated that NfL at v_1_ may be a prognostic indicator in women at normal levels of hsCRP close to the mean and that its value diminishes at higher hsCRP levels. This is a novel finding that was not found in previous studies and requires further replication in larger samples. Our findings among men indicate that when the annual rate of change in NfL is set at its mean, both BMI and hypercholesterolemia may consistently become protective against all-cause mortality. This finding did not apply to women and needs to be replicated in larger studies of the comparable population but with longer follow-up periods.

Here, we have analyzed the longitudinal levels of plasma NfL. Very few studies have examined the longitudinal changes in the NfL levels in relation to health outcomes. Moreover, our study consisted of community-dwelling non-demented participants who are racially and socio-economically diverse. This scope broadens the current knowledge about the usefulness of plasma NfL in the population and also in the absence of neurological disease. Both a strength and a limitation of our study are the age of our study participants. In general, this middle-aged cohort is younger with a lower mean NfL at baseline compared to other studies, which typically are in older adults. However, given that we do observe significant associations with mortality in this younger population leads credence to the weathering and accelerated aging phenotype and premature mortality experienced by urban-dwelling African American and White adults living in Baltimore, MD [53–55]. Therefore, this cohort represents opportunities to identify early biomarkers of mortality that may identify middle-aged individuals that are at risk for premature mortality. This is especially relevant given the current mortality statistics showing a rise in midlife mortality [56]. Because of the baseline age of our cohort and the short follow-up, we are limited in the number of deaths, which may reduce the statistical power to identify additional associations with mortality. Moreover, the small sample size coupled with a limited number of deaths at follow-up reduced statistical power to detect interactive effects by binary effect modifiers with a relatively low prevalence, such as self-reported cardiovascular disease, in the four-way decomposition models, particularly when stratified by sex. Thus, the extrapolation of our findings to the larger target population may be limited, pending further studies with longer follow-up time after the last measure of plasma NfL. Moreover, selection bias due to unequal distribution by factors that were not accounted for compared to the initial sample is a possibility, explaining in part the sex-specific finding for NfL and all-cause mortality. We used 2-stage Heckman selection models to adjust for selection by key socio-demographic factors, including age, sex, race, and poverty status. However, further analysis suggested that women who reported cardio-vascular disease were selected to a greater extent compared to men, which explains the association between NfL change and mortality being restricted to women. Moreover, the role of chance cannot be ruled with multiplicity in hypotheses and mediators as well as effect modifiers. Finally, residual confounding cannot be discounted in explaining some of our key findings.

## Conclusions

In conclusion, we report that plasma NfL levels measured both at baseline and over time can predict all-cause mortality in women. These findings merit further investigation in larger comparable samples of middle-aged adults, which will add to its usefulness as a potential prognostic marker at varying degrees of cardio-metabolic risk, particularly in terms of HbA1c and hsCRP levels. Nevertheless, it is important to identify biomarkers of mortality in middle-aged adults given the alarming recent increase in midlife all-cause mortality [56]. The sex-specific predictive value needs to be examined further both by attempting to replicate these findings in other observational studies and through uncovering the mechanisms behind these differences.

## Supplementary Information

**Additional file 1: Fig. S1.** Participant flowchart illustrating plasma NfL measurements and mortality for this study. *Abbreviations*: δ = Annualized change; HANDLS = Healthy Aging Neighborhoods of Diversity Across the Life Span; NfL = Neurofilament Light Chain. **Method S1.** NfL sample selection. **Table S1.** Allostatic load indicator criteria. **Method S2.** Mixed-effects regression models. **Fig. S2.** Observed vs. empirical bayes estimator for annualized rate of change in Log_e_ transformed NfL, Pearson’s *r* = 0.83, *p* < 0.001. **Table S2.** BMI, AL, AL continuous parameters and cardio-metabolic co-morbidity indices and their relation to all-cause mortality by sex: mediating and moderating effects of NfL_v1_ using 4-way decomposition ^a,b,c^. *Abbreviations*: AL = Allostatic Load; ALB = Albumin; BMI = Body Mass Index; CDE = Controlled Direct Effect; CES-D = Center for Epidemiological Studies-Depression; CHOL = Total cholesterol; CRP = C-reactive protein (high sensitivity), Log_e_ transformed; CVD = Cardiovascular Disease; DBP = Diastolic Blood Pressure; DIAB = Diabetes; HBA1C = Glycated Hemoglobin; HDL = High Density Lipoprotein-Cholesterol; HEI-2010 = Healthy Eating Index-2010 version; HYPERT = Hypertension; HYPERCHOL = Hypercholesterolemia; IM = Interaction, mediated; IR = Interaction, Reference; M = Mediators/Moderator; NfL = Plasma Neurofilament Light Chain, Log_e_ transformed; PM-Pure Mediation; RHR = Resting Heart Rate; SBP = Systolic Blood Pressure; WHR = Waist-Hip Ratio; X = Exposure. ^a^ See Methods and Table 1 for definition of each NfL exposure (i.e., NfLv1 and δNfL). All exposures (X) and potential mediators/moderators (M) were z-scored for ease of interpretation, with the exception of binary M (coded as 0/1), namely DIAB, HYPERT, HYPERCHOL, CVD. Control variables were set at their means. ^b^ Cox models for which 4-way decomposition was conducted is equivalent to Model 2, Table 2, for continuous exposures, to which M was added and considered as a potential mediator/moderator. Control variables included age at v1, sex, race, poverty status, education, HEI-2010 total score, mean energy intake (kcal/d), current tobacco use, current illicit drug use, the CES-D total score and the inverse mills ratio. ^c^ Total effects are beta = Log_e_(HR)±SE with associated p-values from Cox PH hazards models associated with each exposure of interest. Hazard Ratios (HR) point estimates exponent of beta. 95% CI for HR can be calculated as follows: Lower confidence limit, LCL: exp[Log_e_HR-1.96SE(Log_e_HR)], upper confidence limit, UCL: exp[Log_e_HR+1.96SE(Log_e_HR)]. **Table S3.** BMI, AL, AL continuous parameters and cardio-metabolic co-morbidity indices and their relation to all-cause mortality by sex: mediating and moderating effects of δNfL using 4-way decomposition ^a,b,c^. *Abbreviations*: AL = Allostatic Load; ALB = Albumin; BMI = Body Mass Index; CDE = Controlled Direct Effect; CES-D = Center for Epidemiological Studies-Depression; CHOL = Total cholesterol; CRP = C-reactive protein (high sensitivity), Log_e_ transformed; CVD = Cardiovascular Disease; DBP = Diastolic Blood Pressure; DIAB = Diabetes; HBA1C = Glycated Hemoglobin; HDL = High Density Lipoprotein-Cholesterol; HEI-2010 = Healthy Eating Index-2010 version; HYPERT = Hypertension; HYPERCHOL = Hypercholesterolemia; IM = Interaction, mediated; IR = Interaction, Reference; M = Mediators/Moderator; NfL = Plasma Neurofilament Light Chain, Log_e_ transformed; PM-Pure Mediation; RHR = Resting Heart Rate; SBP = Systolic Blood Pressure; WHR = Waist-Hip Ratio; X = Exposure. ^a^ See Methods and Table 1 for definition of each NfL exposure (i.e., NfLv1 and δNfL). All exposures (X) and potential mediators/moderators (M) were z-scored for ease of interpretation, with the exception of binary M (coded as 0/1), namely DIAB, HYPERT, HYPERCHOL, CVD. Control variables were set at their means. ^b^ Cox models for which 4-way decomposition was conducted is equivalent to Model 2, Table 2, for continuous exposures, to which M was added and considered as a potential mediator/moderator. Control variables included age at v1, sex, race, poverty status, education, HEI-2010 total score, mean energy intake (kcal/d), current tobacco use, current illicit drug use, the CES-D total score and the inverse mills ratio. ^c^ Total effects are beta = Log_e_(HR)±SE with associated p-values from Cox PH hazards models associated with each exposure of interest. Hazard Ratios (HR) point estimates exponent of beta. 95% CI for HR can be calculated as follows: Lower confidence limit, LCL: exp[Log_e_HR-1.96SE(Log_e_HR)], upper confidence limit, UCL: exp[Log_e_HR+1.96SE(Log_e_HR)].

## Data Availability

Upon request, data can be made available to researchers with approved proposals, after they have agreed to confidentiality as required by our IRB. Policies are publicized on https://handls.nih.gov. Data access request can be sent to principal investigators (PIs) or the study manager, Jennifer Norbeck at norbeckje@mail.nih.gov. These data are owned by the National Institute on Aging at the NIH. The PIs have made those data restricted to the public for two main reasons: “(1) the study collects medical, psychological, cognitive, and psychosocial information on racial and poverty differences that could be misconstrued or willfully manipulated to promote racial discrimination, and (2) although the sample is fairly large, there are sufficient identifiers that the PIs cannot guarantee absolute confidentiality for every participant as we have stated in acquiring our confidentiality certificate.” Codebook and statistical analysis script can be readily obtained from the corresponding author, upon request, by e-mail contact at baydounm@mail.nih.gov.

## Abbreviations

AD: Alzheimer’s disease
APOE: Apolipoprotein E genotype
BMI: Body mass index
CES-D: Center for Epidemiologic Studies-Depression
CI: Confidence interval
CSF: Cerebrospinal fluid
HANDLS: Healthy Aging in Neighborhoods of Diversity Across the Life Span
HEI-2010: Healthy Eating Index 2010 version
HR: Hazard ratio
HS: High school
IMR: Inverse Mills ratio
IRB: Institutional Review Board
MRV: Medical research vehicles
NfL: Plasma neurofilament light
OLS: Ordinary least squares
SD: Standard deviation
SE: Standard error
US: United States
