# Supplementary material for: Plasma neurofilament light and its association with all-cause mortality risk among urban middle-aged men and women
Source: BMC Med. 2022 Jun 13;20:218. doi: 10.1186/s12916-022-02425-x (PMC9190073; doi:10.1186/s12916-022-02425-x)
Supplement: Supplementary file 1 — Additional file 1: Fig. S1. Participant flowchart illustrating plasma NfL measurements and mortality for this study. Abbreviations: δ = Annualized change; HANDLS = Healthy Aging Neighborhoods of Diversity Across the Life Span; NfL = Neurofilament Light Chain. Method S1. NfL sample selection. Table S1. Allostatic load indicator criteria. Method S2. Mixed-effects regression models. Fig. S2. Observed vs. empirical bayes estimator for annualized rate of change in Loge transformed NfL, Pearson’s r = 0.83, p < 0.001. Table S2. BMI, AL, AL continuous parameters and cardio-metabolic co-morbidity indices and their relation to all-cause mortality by sex: mediating and moderating effects of NfLv1 using 4-way decomposition a,b,c. Abbreviations: AL = Allostatic Load; ALB = Albumin; BMI = Body Mass Index; CDE = Controlled Direct Effect; CES-D = Center for Epidemiological Studies-Depression; CHOL = Total cholesterol; CRP = C-reactive protein (high sensitivity), Loge transformed; CVD = Cardiovascular Disease; DBP = Diastolic Blood Pressure; DIAB = Diabetes; HBA1C = Glycated Hemoglobin; HDL = High Density Lipoprotein-Cholesterol; HEI-2010 = Healthy Eating Index-2010 version; HYPERT = Hypertension; HYPERCHOL = Hypercholesterolemia; IM = Interaction, mediated; IR = Interaction, Reference; M = Mediators/Moderator; NfL = Plasma Neurofilament Light Chain, Loge transformed; PM-Pure Mediation; RHR = Resting Heart Rate; SBP = Systolic Blood Pressure; WHR = Waist-Hip Ratio; X = Exposure. a See Methods and Table 1 for definition of each NfL exposure (i.e., NfLv1 and δNfL). All exposures (X) and potential mediators/moderators (M) were z-scored for ease of interpretation, with the exception of binary M (coded as 0/1), namely DIAB, HYPERT, HYPERCHOL, CVD. Control variables were set at their means. b Cox models for which 4-way decomposition was conducted is equivalent to Model 2, Table 2, for continuous exposures, to which M was added and considered as a potential mediator/moderator. Control vari [file 12916_2022_2425_MOESM1_ESM.docx]

**Plasma Neurofilament Light and its association with all-cause mortality risk among urban middle-aged men and women**

**By Beydoun, M. A. et al.**

**Fig S1.** Participant flowchart illustrating plasma NfL measurements and mortality for this study

**Final sample:**

**NfL visit 1 and δNfL**

**N=694 (401 women, 293 men)**

Mortality follow-up;

Mean follow-up time: 11.2 years

(range: 3.86-14.31)

**All-cause deaths through 2018 (N=43):**

Women (N=20) and Men (N=23)

NfL visit 2

NfL visit 1

NfL visit 3

NfL available for any of 3 visits;

N=731

**δNfL: Annualized change in NfL**

Mean follow-up time: 7.77 years (range: 4.9-12.5)

*Abbreviations*: δ=Annualized change; HANDLS=Healthy Aging Neighborhoods of Diversity Across the Life Span; NfL=Neurofilament Light Chain.

**Method S1: NfL sample selection**

Plasma NfL was quantified in a sub-cohort of participants from HANDLS from visits v_1_ (2004-2009), v_2_ (2009-2013) and v_3_ (2013-2017), all of which were used for our present study. This sub-sample included participants from the HANDLS SCAN, an ancillary neuroimaging sub-study, (n=238)[1] This sub-study of the HANDLS cohort excluded participants with a history of dementia, stroke, transient ischemic attack, and carotid endarterectomy, MRI contraindications, terminal illness, HIV positivity or other neurological disorders [1]. All HANDLS SCAN participants included in this sub-study had donated plasma samples at three different visits except for one participant that had samples from only 2 of 3 visits. In addition, we also included participants (n=463; 1389 samples) that donated plasma samples at v_1_, v_2_ and v_3_, who were HIV negative, had complete cognitive tests [Trailmaking test, part A (TRAILS A) and Digits Span-Forward (DS-F)[ at v_1_ and v_2_, Centers of Epidemiologic Studies-Depression (CES-D) scores at all 3 visits and with no history of HIV, stroke, transient ischemic attack, dementia, epilepsy, Parkinson’s disease or brain cancer. Participants (n=3) were also included who had plasma samples available from v_1_, v_2_ and v_3_, who also had genome wide DNA methylation data at v_1_[2-4]. These participants had the exclusions listed above. Thus, overall, N=694 HANDLS participants had plasma NfL data at v_1_ ; N=709 at v_2_ and N=707.

**Table S1.** Allostatic load indicator criteria[5].

|  | **High-risk clinical** |
| --- | --- |
| Waist:Hip | >0.9 for men; > 0.85 for women [6] |
| Albumin (g/dL) | < 3.8 [7] |
| C-reactive protein (mg/dL) | ≥ 0.3 [8] |
| Total cholesterol (mg/dL) | ≥240[9] |
| HDL (mg/dL) | <40[9] |
| Glycated hemoglobin (%) | ≥6.4[10, 11] |
| Resting heart rate (beat/min) | ≥90[12] |
| Systolic BP | ≥140[13] |
| Diastolic BP | ≥90[13] |

**Method S2**: Mixed-effects regression models

The main multiple mixed-effects regression models can be summarized as follows:

**Multi-level models** vs. **Composite models**

| **Eq.**  **1.1-1.4** |  |  |  |
| --- | --- | --- | --- |

Where Y_ij_ is the outcome (plasma NfL measured at v_1_ , v_2_ and/or v_3_) for each individual “i” and visit “j”; is the level-1 intercept for individual i; is the level-1 slope for individual i; is the level-2 intercept of the random intercept ; is the level-2 intercept of the slope ; is a vector of fixed covariates for each individual *i* that are used to predict level-1 intercepts and slopes, which can include socio-demographic variables among others. In this analysis, mixed-effects regression models included AL total score exposure measured at v_1_ (X_ij_) along with covariates (Z_ij_). and are level-2 disturbances; is the within-person level-1 disturbance [14].

It is worth noting that the models were fit using the entire HANDLS cohort with complete data on either v_1_, v_2_ or v_3_ on NfL was used to improve reliability of predicted estimates. Empirical bayes estimators for annual rate of change in NfL (δNfL) were also predicted from time-interval mixed-effects models, with up to 3 repeats on plasma NfL as the outcome and covariates between v_1_ Age, sex, race and poverty status. This estimate was used as a validation tool, against observed annualized change in NfL between those 3 visits. The latter was computed as the arithmetic mean in the annualized changes of Log_e_ transformed NfL between v1 and v2; v2 and v3; and v1 and v3. The individual-level observed annualized rate of change (δNfL_obs_) depended on 1, 2 or 3 values of annualized changes and thus there was no additional missing data for this estimate. The scatter plot of δNfLobs and δNfLbayes is shown in **Fig S2.**

**Fig S2.** Observed vs. empirical bayes estimator for annualized rate of change in Log_e_ transformed NfL, Pearson’s r=0.83, p<0.001

**Supplemental References**

1. Waldstein SR, Dore GA, Davatzikos C, Katzel LI, Gullapalli R, Seliger SL, Kouo T, Rosenberger WF, Erus G, Evans MK *et al*: **Differential Associations of Socioeconomic Status With Global Brain Volumes and White Matter Lesions in African American and White Adults: the HANDLS SCAN Study**. *Psychosom Med* 2017, **79**(3):327-335.

2. Beydoun MA, Hossain S, Chitrala KN, Tajuddin SM, Beydoun HA, Evans MK, Zonderman AB: **Association between epigenetic age acceleration and depressive symptoms in a prospective cohort study of urban-dwelling adults**. *J Affect Disord* 2019, **257**:64-73.

3. Beydoun MA, Shaked D, Tajuddin SM, Weiss J, Evans MK, Zonderman AB: **Accelerated epigenetic age and cognitive decline among urban-dwelling adults**. *Neurology* 2020, **94**(6):e613-e625.

4. Tajuddin SM, Hernandez DG, Chen BH, Noren Hooten N, Mode NA, Nalls MA, Singleton AB, Ejiogu N, Chitrala KN, Zonderman AB *et al*: **Novel age-associated DNA methylation changes and epigenetic age acceleration in middle-aged African Americans and whites**. *Clin Epigenetics* 2019, **11**(1):119.

5. Seeman T, Merkin SS, Crimmins E, Koretz B, Charette S, Karlamangla A: **Education, income and ethnic differences in cumulative biological risk profiles in a national sample of US adults: NHANES III (1988-1994)**. *Social science & medicine* 2008, **66**(1):72-87.

6. Alberti KG, Zimmet PZ: **Definition, diagnosis and classification of diabetes mellitus and its complications. Part 1: diagnosis and classification of diabetes mellitus provisional report of a WHO consultation**. *Diabetic medicine : a journal of the British Diabetic Association* 1998, **15**(7):539-553.

7. Visser M, Kritchevsky SB, Newman AB, Goodpaster BH, Tylavsky FA, Nevitt MC, Harris TB: **Lower serum albumin concentration and change in muscle mass: the Health, Aging and Body Composition Study**. *Am J Clin Nutr* 2005, **82**(3):531-537.

8. Ridker PM: **Cardiology Patient Page. C-reactive protein: a simple test to help predict risk of heart attack and stroke**. *Circulation* 2003, **108**(12):e81-85.

9. Expert Panel on Detection E, Treatment of High Blood Cholesterol in A: **Executive Summary of The Third Report of The National Cholesterol Education Program (NCEP) Expert Panel on Detection, Evaluation, And Treatment of High Blood Cholesterol In Adults (Adult Treatment Panel III)**. *Jama* 2001, **285**(19):2486-2497.

10. Golden S, Boulware LE, Berkenblit G, Brancati F, Chander G, Marinopoulos S, Paasche-Orlow M, Powe N, Rami T: **Use of glycated hemoglobin and microalbuminuria in the monitoring of diabetes mellitus**. *Evidence report/technology assessment* 2003(84):1-6.

11. Osei K, Rhinesmith S, Gaillard T, Schuster D: **Is glycosylated hemoglobin A1c a surrogate for metabolic syndrome in nondiabetic, first-degree relatives of African-American patients with type 2 diabetes?** *The Journal of clinical endocrinology and metabolism* 2003, **88**(10):4596-4601.

12. Seccareccia F, Pannozzo F, Dima F, Minoprio A, Menditto A, Lo Noce C, Giampaoli S, Malattie Cardiovascolari Aterosclerotiche Istituto Superiore di Sanita P: **Heart rate as a predictor of mortality: the MATISS project**. *American journal of public health* 2001, **91**(8):1258-1263.

13. Lenfant C, Chobanian AV, Jones DW, Roccella EJ, Joint National Committee on the Prevention DE, Treatment of High Blood P: **Seventh report of the Joint National Committee on the Prevention, Detection, Evaluation, and Treatment of High Blood Pressure (JNC 7): resetting the hypertension sails**. *Hypertension* 2003, **41**(6):1178-1179.

14. Blackwell E, de Leon CF, Miller GE: **Applying mixed regression models to the analysis of repeated-measures data in psychosomatic medicine**. *Psychosom Med* 2006, **68**(6):870-878.

**Table S2**. BMI, AL, AL continuous parameters and cardio-metabolic co-morbidity indices and their relation to all-cause mortality by sex: mediating and moderating effects of NfL_v1_ using 4-way decomposition ^a,b,c^

|  |  | Overall (N=694) |  | Women (N=401) |  | Men  (N=293) |  |
| --- | --- | --- | --- | --- | --- | --- | --- |
|  |  | β±SE | P | β±SE | P | β±SE | P |
| **X=BMI; M=NfL_v1_** |  |  |  |  |  |  |  |
| Total effect |  | -0.108±0.183 | 0.554 | +0.126±0.322 | 0.696 | -0.391±0.257 | 0.128 |
| CDE |  | -0.128±0.168 | 0.445 | +0.197±0.322 | 0.541 | **-0.441±0.196** | **0.024** |
| IR |  | -0.004±0.041 | 0.930 | -0.067±0.084 | 0.429 | +0.013±0.051 | 0.805 |
| IM |  | +0.056±0.035 | 0.108 | +0.098±0.061 | 0.108 | +0.015±0.048 | 0.764 |
| PM |  | -0.033±0.039 | 0.396 | -0.102±0.054 | 0.058 | +0.022±0.076 | 0.767 |
| **X=AL; M=NfL_v1_** |  |  |  |  |  |  |  |
| Total effect |  | +0.018±0.175 | 0.917 | +0.163±0.326 | 0.618 | -0.098±0.226 | 0.664 |
| CDE |  | +0.038±0.179 | 0.831 | +0.209±0.336 | 0.534 | -0.128±0.222 | 0.564 |
| IR |  | -0.007±0.022 | 0.762 | -0.024±0.053 | 0.644 | +0.007±0.065 | 0.909 |
| IM |  | +0.002±0.009 | 0.798 | +0.001±0.008 | 0.872 | +0.022±0.024 | 0.363 |
| PM |  | -0.016±0.014 | 0.246 | -0.023±0.028 | 0.408 | +0.001±0.027 | 0.969 |
| **X=WHR; M=NfL_v1_** |  |  |  |  |  |  |  |
| Total effect |  | +0.226±2.319 | 0.923 | -0.359±1.115 | 0.747 | +0.894±12.998 | 0.945 |
| CDE |  | +0.073±1.581 | 0.964 | -0.363±1.158 | 0.754 | -0.513±0.916 | 0.577 |
| IR |  | +0.187±1.215 | 0.878 | -0.017±0.177 | 0.923 | +0.484±3.677 | 0.895 |
| IM |  | -0.047±0.167 | 0.777 | -0.012±0.044 | 0.779 | +0.963±9.141 | 0.916 |
| PM |  | +0.014±0.013 | 0.279 | +0.033±0.024 | 0.165 | -0.039±0.188 | 0.833 |
| **X=ALB; M=NfL_v1_** |  |  |  |  |  |  |  |
| Total effect |  | +0.083±0.211 | 0.696 | -0.023±0.302 | 0.939 | +0.044±0.289 | 0.878 |
| CDE |  | +0.103±0.208 | 0.621 | -0.055±0.313 | 0.860 | +0.003±0.253 | 0.992 |
| IR |  | -0.014±0.018 | 0.437 | +0.05±0.087 | 0.562 | -0.007±0.058 | 0.907 |
| IM |  | +0.012±0.016 | 0.453 | -0.004±0.010 | 0.683 | +0.047±0.044 | 0.283 |
| PM |  | -0.018±0.016 | 0.270 | -0.014±0.024 | 0.553 | +0.001±0.047 | 0.978 |
| **X=CRP; M=NfL_v1_** |  |  |  |  |  |  |  |
| Total effect |  | +0.171±0.216 | 0.428 | +0.600±0.502 | 0.231 | +0.194±0.338 | 0.566 |
| CDE |  | +0.214±0.219 | 0.328 | +0.802±0.522 | 0.125 | +0.164±0.288 | 0.568 |
| IR |  | -0.026±0.025 | 0.304 | -0.169±0.106 | 0.110 | +0.008±0.105 | 0.941 |
| IM |  | +0.010±0.015 | 0.506 | +0.030±0.030 | 0.315 | +0.025±0.034 | 0.457 |
| PM |  | -0.027±0.018 | 0.131 | -0.062±0.041 | 0.128 | -0.003±0.022 | 0.882 |
| **X=HBAIC; M=NfL_v1_** |  |  |  |  |  |  |  |
| Total effect |  | +0.158±0.173 | 0.360 | +0.290±0.234 | 0.216 | +0.030±0.274 | 0.914 |
| CDE |  | +0.145±0.178 | 0.416 | +0.261±0.228 | 0.252 | +0.040±0.284 | 0.889 |
| IR |  | -0.001±0.014 | 0.932 | -0.012±0.026 | 0.642 | -0.010±0.027 | 0.715 |
| IM |  | +0.001±0.005 | 0.919 | +0.002±0.008 | 0.765 | -0.004±0.012 | 0.746 |
| PM |  | +0.014±0.014 | 0.288 | +0.039±0.033 | 0.238 | +0.004±0.013 | 0.771 |
| **X=CHOL; M=NfL_v1_** |  |  |  |  |  |  |  |
| Total effect |  | -0.050±0.166 | 0.763 | +0.194±0.315 | 0.538 | -0.223±0.194 | 0.252 |
| CDE |  | -0.048±0.172 | 0.782 | +0.194±0.325 | 0.549 | -0.230±0.197 | 0.242 |
| IR |  | +0.014±0.026 | 0.598 | +0.006±0.044 | 0.894 | +0.032±0.064 | 0.620 |
| IM |  | -0.004±0.007 | 0.597 | -0.001±0.004 | 0.825 | -0.019±0.026 | 0.470 |
| PM |  | -0.012±0.012 | 0.306 | -0.006±0.022 | 0.800 | -0.005±0.032 | 0.868 |
| **X=HDL; M=NfL_v1_** |  |  |  |  |  |  |  |
| Total effect |  | +0.274±0.199 | 0.170 | +0.522±0.378 | 0.168 | +0.167±0.270 | 0.536 |
| CDE |  | +0.129±0.199 | 0.517 | +0.259±0.356 | 0.467 | +0.132±0.285 | 0.643 |
| IR |  | +0.080±0.083 | 0.336 | +0.166±0.173 | 0.336 | +0.014±0.097 | 0.886 |
| IM |  | +0.037±0.027 | 0.180 | +0.055±0.049 | 0.258 | +0.010±0.060 | 0.866 |
| PM |  | +0.027±0.021 | 0.204 | +0.041±0.033 | 0.209 | +0.011±0.044 | 0.798 |
| **X=RHR; M=NfL_v1_** |  |  |  |  |  |  |  |
| Total effect |  | +0.327±0.216 | 0.130 | +0.496±0.440 | 0.261 | +0.292±0.279 | 0.296 |
| CDE |  | +0.356±0.224 | 0.113 | +0.539±0.456 | 0.239 | +0.327±0.280 | 0.245 |
| IR |  | -0.021±0.030 | 0.484 | 0.000±0.051 | 0.997 | -0.037±0.103 | 0.718 |
| IM |  | +0.003±0.007 | 0.663 | -0.010±0.015 | 0.517 | +0.004±0.025 | 0.867 |
| PM |  | -0.010±0.012 | 0.399 | -0.033±0.030 | 0.283 | -0.002±0.012 | 0.843 |
| **X=SBP; M=NfL_v1_** |  |  |  |  |  |  |  |
| Total effect |  | +0.040±0.186 | 0.832 | -0.015±0.259 | 0.955 | +0.063±0.299 | 0.833 |
| CDE |  | +0.059±0.192 | 0.758 | +0.055±0.276 | 0.843 | +0.051±0.297 | 0.865 |
| IR |  | +0.007±0.023 | 0.767 | -0.004±0.030 | 0.896 | +0.026±0.072 | 0.722 |
| IM |  | -0.005±0.012 | 0.708 | 0.000±0.016 | 0.987 | -0.010±0.025 | 0.680 |
| PM |  | -0.022±0.019 | 0.248 | -0.065±0.038 | 0.089 | -0.003±0.020 | 0.875 |
| **X=DBP; M=NfL_v1_** |  |  |  |  |  |  |  |
| Total effect |  | +0.181±0.209 | 0.387 | +0.150±0.317 | 0.637 | +0.171±0.288 | 0.553 |
| CDE |  | +0.205±0.215 | 0.340 | +0.203±0.340 | 0.549 | +0.182±0.294 | 0.537 |
| IR |  | -0.002±0.030 | 0.947 | +0.078±0.120 | 0.513 | -0.009±0.045 | 0.837 |
| IM |  | -0.001±0.015 | 0.960 | -0.048±0.050 | 0.342 | +0.001±0.007 | 0.880 |
| PM |  | -0.021±0.017 | 0.200 | -0.084±0.044 | 0.055 | -0.002±0.008 | 0.792 |
| **X=HYPERT; M=NfL_v1_** |  |  |  |  |  |  |  |
| Total effect |  | +0.412±0.531 | 0.438 | +1.772±1.674 | 0.290 | -0.190±0.425±± | 0.655 |
| CDE |  | +0.321±0.522 | 0.538 | +1.487±1.554 | 0.339 | -0.164±0.438±± | 0.708 |
| IR |  | +0.069±0.081 | 0.397 | +0.224±0.282 | 0.426 | -0.021±0.063±± | 0.732 |
| IM |  | +0.018±0.037 | 0.627 | +0.056±0.158 | 0.723 | -0.012±0.038± | 0.740 |
| PM |  | +0.003±0.012 | 0.771 | +0.005±0.021 | 0.822 | +0.008±0.024± | 0.747 |
| **X=DIAB; M=NfL_v1_** |  |  |  |  |  |  |  |
| Total effect |  | +0.249±0.442 | 0.574 | +1.265±1.294 | 0.328 | -0.251±0.373 | 0.501 |
| CDE |  | +0.337±0.455 | 0.460 | +1.477±1.367 | 0.280 | -0.209±0.378 | 0.580 |
| IR |  | -0.074±0.077 | 0.335 | -0.048±0.183 | 0.792 | -0.058±0.115 | 0.613 |
| IM |  | +0.047±0.055 | 0.395 | -0.038±0.143 | 0.791 | +0.038±0.068 | 0.576 |
| PM |  | -0.060±0.043 | 0.161 | -0.126±0.082 | 0.126 | -0.022±0.042 | 0.604 |
| **X=HYPERCHOL; M=NfL_v1_** |  |  |  |  |  |  |  |
| Total effect |  | -0.342±0.324 | 0.294 | -0.123±0.574 | 0.831 | ***-0.643±0.346*** | ***0.074*** |
| CDE |  | -0.343±0.330 | 0.301 | -0.131±0.608 | 0.830 | ***-0.619±0.351*** | ***0.087*** |
| IR |  | +0.014±0.068 | 0.838 | +0.072±0.123 | 0.556 | -0.024±0.071 | 0.736 |
| IM |  | -0.004±0.020 | 0.834 | -0.023±0.052 | 0.660 | -0.002±0.033 | 0.941 |
| PM |  | -0.009±0.022 | 0.676 | -0.041±0.052 | 0.430 | +0.002±0.026 | 0.932 |
| **X=CVD; M=NfL_v1_** |  |  |  |  |  |  |  |
| Total effect |  | -0.483±0.328 | 0.141 | **-0.601±0.364** | **0.099** | -0.231±0.791 | 0.770 |
| CDE |  | -0.694±0.266 | 0.009 | **-0.803±0.249** | **0.001** | -0.588±0.553 | 0.290 |
| IR |  | +0.205±0.126 | 0.105 | +0.193±0.160 | 0.229 | +0.372±0.730 | 0.610 |
| IM |  | +0.005±0.057 | 0.933 | +0.006±0.055 | 0.911 | -0.016±0.202 | 0.939 |
| PM |  | +0.001±0.009 | 0.882 | +0.003±0.026 | 0.921 | 0.000±0.013 | 0.988 |
|  |  |  |  |  |  |  |  |

*Abbreviations*: AL=Allostatic Load; ALB=Albumin; BMI=Body Mass Index; CDE=Controlled Direct Effect; CES-D=Center for Epidemiological Studies-Depression; CHOL=Total cholesterol; CRP=C-reactive protein (high sensitivity), Log_e_ transformed; CVD=Cardiovascular Disease; DBP=Diastolic Blood Pressure; DIAB=Diabetes; HBA1C=Glycated Hemoglobin; HDL=High Density Lipoprotein-Cholesterol; HEI-2010=Healthy Eating Index-2010 version; HYPERT=Hypertension; HYPERCHOL=Hypercholesterolemia; IM=Interaction, mediated; IR=Interaction, Reference; M=Mediators/Moderator; NfL= Plasma Neurofilament Light Chain, Log_e_ transformed; PM-Pure Mediation; RHR=Resting Heart Rate; SBP=Systolic Blood Pressure; WHR=Waist-Hip Ratio; X=Exposure.

^a^ See methods and Table 1 for definition of each NfL exposure (i.e. NfLv1 and δNfL). All exposures (X) and potential mediators/moderators (M) were z-scored for ease of interpretation, with the exception of binary M (coded as 0/1), namely DIAB, HYPERT, HYPERCHOL, CVD. Control variables were set at their means.

^b^ Cox models for which 4-way decomposition was conducted is equivalent to Model 2, Table 2, for continuous exposures, to which M was added and considered as a potential mediator/moderator. Control variables included age at v1, sex, race, poverty status, education, HEI-2010 total score, mean energy intake (kcal/d), current tobacco use, current illicit drug use, the CES-D total score and the inverse mills ratio.

^c^ Total effects are beta=Log_e_(HR)±SE with associated p-values from Cox PH hazards models associated with each exposure of interest. Hazard Ratios (HR) point estimates exponent of beta. 95% CI for HR can be calculated as follows: Lower confidence limit, LCL: exp[Log_e_HR-1.96SE(Log_e_HR)], upper confidence limit, UCL: exp[Log_e_HR+1.96SE(Log_e_HR)].

**Table S3**. BMI, AL, AL continuous parameters and cardio-metabolic co-morbidity indices and their relation to all-cause mortality by sex: mediating and moderating effects of δNfL using 4-way decomposition ^a,b,c^

|  |  | Overall (N=694) |  | Women (N=401) |  | Men  (N=293) |  |
| --- | --- | --- | --- | --- | --- | --- | --- |
|  |  | β±SE | P | β±SE | P | β±SE | P |
| **X=BMI; M= δNfL** |  |  |  |  |  |  |  |
| Total effect |  | -0.231±0.145 | 0.110 | -0.171±0.235 | 0.466 | **-0.429±0.204** | **0.036** |
| CDE |  | **-0.301±0.141** | **0.033** | -0.293±0.207 | 0.156 | **-0.484±0.176** | **0.006** |
| IR |  | +0.036±0.038 | 0.344 | +0.044±0.069 | 0.524 | +0.039±0.074 | 0.596 |
| IM |  | +0.009±0.013 | 0.502 | +0.005±0.031 | 0.886 | +0.007±0.015 | 0.654 |
| PM |  | +0.026±0.018 | 0.158 | +0.073±0.042 | 0.081 | +0.009±0.019 | 0.644 |
| **X=AL; M= δNfL** |  |  |  |  |  |  |  |
| Total effect |  | +0.015±0.168 | 0.930 | +0.217±0.314 | 0.489 | -0.156±0.217 | 0.473 |
| CDE |  | -0.001±0.168 | 0.993 | +0.122±0.31 | 0.693 | -0.153±0.213 | 0.474 |
| IR |  | -0.003±0.026 | 0.909 | +0.016±0.069 | 0.814 | -0.004±0.026 | 0.869 |
| IM |  | -0.002±0.016 | 0.902 | +0.014±0.037 | 0.709 | -0.003±0.017 | 0.845 |
| PM |  | +0.021±0.017 | 0.216 | +0.065±0.043 | 0.136 | +0.005±0.018 | 0.782 |
| **X=WHR; M= δNfL** |  |  |  |  |  |  |  |
| Total effect |  | +0.323±2.038 | 0.875 | -0.165±1.666 | 0.921 | +2.517±25.227 | 0.921 |
| CDE |  | -0.035±1.244 | 0.977 | -0.142±1.45 | 0.922 | -0.556±0.698 | 0.427 |
| IR |  | +0.411±1.251 | 0.743 | -0.016±0.509 | 0.974 | +0.897±3.585 | 0.803 |
| IM |  | -0.064±0.155 | 0.681 | -0.028±0.081 | 0.730 | +2.143±22.698 | 0.925 |
| PM |  | +0.011±0.011 | 0.308 | +0.022±0.02 | 0.287 | +0.033±0.156 | 0.835 |
| **X=ALB; M= δNfL** |  |  |  |  |  |  |  |
| Total effect |  | +0.126±0.22 | 0.568 | -0.043±0.297 | 0.884 | +0.362±0.504 | 0.473 |
| CDE |  | +0.104±0.196 | 0.596 | -0.017±0.299 | 0.953 | +0.08±0.262 | 0.761 |
| IR |  | +0.026±0.076 | 0.734 | -0.027±0.049 | 0.574 | +0.312±0.379 | 0.41 |
| IM |  | -0.005±0.014 | 0.692 | -0.001±0.006 | 0.934 | -0.026±0.061 | 0.677 |
| PM |  | +0.001±0.004 | 0.748 | +0.002±0.022 | 0.921 | -0.004±0.011 | 0.717 |
| **X=CRP; M= δNfL** |  |  |  |  |  |  |  |
| Total effect |  | +0.144±0.201 | 0.473 | +0.372±0.419 | 0.375 | +0.104±0.271 | 0.701 |
| CDE |  | +0.153±0.203 | 0.451 | +0.411±0.417 | 0.324 | +0.098±0.268 | 0.714 |
| IR |  | -0.009±0.014 | 0.537 | -0.067±0.067 | 0.314 | -0.001±0.01 | 0.904 |
| IM |  | 0.000±0.003 | 0.875 | -0.016±0.026 | 0.537 | +0.004±0.019 | 0.835 |
| PM |  | +0.001±0.006 | 0.869 | +0.043±0.033 | 0.189 | +0.003±0.02 | 0.880 |
| **X=HBAIC; M= δNfL** |  |  |  |  |  |  |  |
| Total effect |  | -0.193±0.158 | 0.224 | -0.165±0.301 | 0.584 | -0.210±0.205 | 0.307 |
| CDE |  | -0.334±0.177 | 0.059 | -0.309±0.328 | 0.346 | -0.363±0.216 | 0.093 |
| IR |  | **+0.082±0.041** | **0.047** | +0.065±0.050 | 0.190 | +0.103±0.080 | 0.199 |
| IM |  | **+0.037±0.015** | **0.012** | +0.020±0.020 | 0.315 | +0.049±0.028 | 0.076 |
| PM |  | +0.022±0.021 | 0.30 | +0.058±0.043 | 0.180 | +0.002±0.03 | 0.954 |
| **X=CHOL; M= δNfL** |  |  |  |  |  |  |  |
| Total effect |  | -0.018±0.155 | 0.909 | +0.203±0.288 | 0.480 | -0.155±0.209 | 0.457 |
| CDE |  | -0.134±0.144 | 0.353 | +0.044±0.263 | 0.868 | -0.217±0.179 | 0.224 |
| IR |  | +0.079±0.066 | 0.235 | +0.098±0.117 | 0.399 | +0.041±0.090 | 0.650 |
| IM |  | +0.017±0.015 | 0.234 | +0.027±0.032 | 0.387 | +0.009±0.017 | 0.602 |
| PM |  | +0.020±0.015 | 0.190 | +0.033±0.028 | 0.232 | +0.013±0.021 | 0.551 |
| **X=HDL; M= δNfL** |  |  |  |  |  |  |  |
| Total effect |  | +0.297±0.193 | 0.123 | +0.598±0.347 | 0.085 | +0.154±0.258 | 0.550 |
| CDE |  | +0.291±0.192 | 0.129 | +0.651±0.340 | 0.056 | +0.163±0.259 | 0.531 |
| IR |  | +0.002±0.031 | 0.953 | -0.038±0.113 | 0.736 | -0.003±0.034 | 0.920 |
| IM |  | +0.001±0.004 | 0.850 | -0.001±0.011 | 0.923 | -0.005±0.015 | 0.723 |
| PM |  | +0.004±0.008 | 0.658 | -0.014±0.031 | 0.656 | 0.000±0.009 | 0.997 |
| **X=RHR; M= δNfL** |  |  |  |  |  |  |  |
| Total effect |  | +0.299±0.210 | 0.156 | +0.426±0.393 | 0.280 | +0.315±0.32 | 0.327 |
| CDE |  | +0.298±0.217 | 0.172 | +0.287±0.383 | 0.456 | +0.341±0.319 | 0.288 |
| IR |  | -0.013±0.020 | 0.509 | +0.053±0.079 | 0.501 | +0.005±0.084 | 0.950 |
| IM |  | -0.011±0.020 | 0.581 | +0.039±0.043 | 0.366 | -0.038±0.04 | 0.337 |
| PM |  | +0.026±0.020 | 0.203 | +0.047±0.041 | 0.251 | +0.007±0.02 | 0.746 |
| **X=SBP; M= δNfL** |  |  |  |  |  |  |  |
| Total effect |  | +0.090±0.180 | 0.617 | +0.076±0.277 | 0.784 | +0.064±0.285 | 0.822 |
| CDE |  | +0.047±0.179 | 0.794 | +0.008±0.238 | 0.972 | +0.023±0.285 | 0.935 |
| IR |  | +0.014±0.029 | 0.631 | +0.019±0.088 | 0.831 | +0.018±0.041 | 0.655 |
| IM |  | +0.010±0.017 | 0.584 | +0.006±0.028 | 0.833 | +0.027±0.043 | 0.532 |
| PM |  | +0.020±0.019 | 0.294 | +0.043±0.032 | 0.178 | -0.004±0.041 | 0.916 |
| **X=DBP; M= δNfL** |  |  |  |  |  |  |  |
| Total effect |  | +0.207±0.210 | 0.324 | +0.321±0.353 | 0.362 | +0.160±0.283 | 0.571 |
| CDE |  | +0.206±0.215 | 0.340 | +0.328±0.351 | 0.349 | +0.136±0.289 | 0.638 |
| IR |  | -0.011±0.026 | 0.658 | -0.023±0.080 | 0.768 | +0.011±0.039 | 0.777 |
| IM |  | -0.004±0.013 | 0.741 | -0.001±0.013 | 0.931 | +0.016±0.039 | 0.680 |
| PM |  | +0.017±0.015 | 0.237 | +0.018±0.028 | 0.521 | -0.003±0.033 | 0.928 |
| **X=HYPERT; M= δNfL** |  |  |  |  |  |  |  |
| Total effect |  | +0.545±0.554 | 0.326 | +2.232±1.889 | 0.237 | -0.217±0.410 | 0.597 |
| CDE |  | +0.539±0.561 | 0.336 | +1.901±1.752 | 0.278 | -0.159±0.432 | 0.713 |
| IR |  | -0.022±0.045 | 0.625 | +0.038±0.193 | 0.842 | -0.009±0.103 | 0.930 |
| IM |  | -0.058±0.095 | 0.545 | +0.214±0.276 | 0.439 | -0.163±0.168 | 0.332 |
| PM |  | +0.085±0.068 | 0.208 | +0.079±0.110 | 0.475 | +0.114±0.124 | 0.358 |
| **X=DIAB; M= δNfL** |  |  |  |  |  |  |  |
| Total effect |  | +0.078±0.379 | 0.838 | +0.860±1.018 | 0.398 | -0.337±0.330 | 0.306 |
| CDE |  | -0.027±0.365 | 0.941 | +0.327±0.862 | 0.704 | -0.368±0.321 | 0.253 |
| IR |  | +0.020±0.038 | 0.598 | +0.172±0.222 | 0.437 | -0.007±0.083 | 0.932 |
| IM |  | +0.075±0.079 | 0.340 | +0.302±0.289 | 0.296 | +0.085±0.106 | 0.423 |
| PM |  | +0.009±0.059 | 0.874 | +0.058±0.108 | 0.590 | -0.047±0.092 | 0.610 |
| **X=HYPERCHOL; M= δNfL** |  |  |  |  |  |  |  |
| Total effect |  | -0.372±0.311 | 0.237 | -0.068±0.553 | 0.902 | -0.661±0.338 | 0.064 |
| CDE |  | -0.496±0.286 | 0.087 | -0.346±0.476 | 0.467 | **-0.684±0.312** | **0.037** |
| IR |  | +0.094±0.098 | 0.339 | +0.159±0.184 | 0.388 | +0.022±0.090 | 0.810 |
| IM |  | +0.022±0.043 | 0.614 | +0.071±0.110 | 0.521 | +0.001±0.030 | 0.985 |
| PM |  | +0.008±0.017 | 0.637 | +0.049±0.061 | 0.425 | +0.001±0.014 | 0.954 |
| **X=CVD; M= δNfL** |  |  |  |  |  |  |  |
| Total effect |  | -0.191±0.437 | 0.663 | -0.373±0.482 | 0.439 | -0.218±0.675 | 0.747 |
| CDE |  | -0.435±0.383 | 0.256 | -0.574±0.431 | 0.183 | -0.290±0.701 | 0.679 |
| IR |  | +0.137±0.132 | 0.301 | +0.156±0.186 | 0.403 | +0.019±0.129 | 0.881 |
| IM |  | +0.082±0.089 | 0.358 | +0.018±0.059 | 0.761 | +0.023±0.370 | 0.951 |
| PM |  | +0.026±0.035 | 0.460 | +0.026±0.055 | 0.632 | +0.030±0.129 | 0.815 |
|  |  |  |  |  |  |  |  |

*Abbreviations*: AL=Allostatic Load; ALB=Albumin; BMI=Body Mass Index; CDE=Controlled Direct Effect; CES-D=Center for Epidemiological Studies-Depression; CHOL=Total cholesterol; CRP=C-reactive protein (high sensitivity), Log_e_ transformed; CVD=Cardiovascular Disease; DBP=Diastolic Blood Pressure; DIAB=Diabetes; HBA1C=Glycated Hemoglobin; HDL=High Density Lipoprotein-Cholesterol; HEI-2010=Healthy Eating Index-2010 version; HYPERT=Hypertension; HYPERCHOL=Hypercholesterolemia; IM=Interaction, mediated; IR=Interaction, Reference; M=Mediators/Moderator; NfL= Plasma Neurofilament Light Chain, Log_e_ transformed; PM-Pure Mediation; RHR=Resting Heart Rate; SBP=Systolic Blood Pressure; WHR=Waist-Hip Ratio; X=Exposure.

^a^ See methods and Table 1 for definition of each NfL exposure (i.e. NfLv1 and δNfL). All exposures (X) and potential mediators/moderators (M) were z-scored for ease of interpretation, with the exception of binary M (coded as 0/1), namely DIAB, HYPERT, HYPERCHOL, CVD. Control variables were set at their means.

^b^ Cox models for which 4-way decomposition was conducted is equivalent to Model 2, Table 2, for continuous exposures, to which M was added and considered as a potential mediator/moderator. Control variables included age at v1, sex, race, poverty status, education, HEI-2010 total score, mean energy intake (kcal/d), current tobacco use, current illicit drug use, the CES-D total score and the inverse mills ratio.

^c^ Total effects are beta=Log_e_(HR)±SE with associated p-values from Cox PH hazards models associated with each exposure of interest. Hazard Ratios (HR) point estimates exponent of beta. 95% CI for HR can be calculated as follows: Lower confidence limit, LCL: exp[Log_e_HR-1.96SE(Log_e_HR)], upper confidence limit, UCL: exp[Log_e_HR+1.96SE(Log_e_HR)].
